# Thermo-mechanical behavior and spalling resistance of alkali-activated slag versus cement mortars under rapid high-temperature exposure

**DOI:** 10.1038/s41598-025-19301-2

**Published:** 2025-09-17

**Authors:** A. Y. F. Ali, Sabry A. Ahmed, Y. H. Helal, M. S. El-Feky

**Affiliations:** 1 Materials Engineering Department, Zagazig University, Zagazig, 44519 Egypt; 2 Higher Technological Institute, 10TH of Ramadan City, Egypt; 3 Department of Civil Engineering, National Research Centre, Cairo, Egypt

**Keywords:** Alkali-activated slag mortar, High-strength cement mortar, Elevated temperature exposure, Fire-induced spalling, Thermal insulation, Residual mechanical properties, Microstructural characterization, Engineering, Materials science

## Abstract

The susceptibility of high-strength cementitious composites to explosive spalling under elevated temperatures necessitates the development of sustainable, fire-resistant alternatives for structural applications. This study comparatively evaluates the thermo-mechanical performance and spalling resistance of high-strength alkali-activated slag mortar (HSAAM) and ordinary Portland cement-based mortar (HSCM) under rapid fire scenarios. HSAAM was synthesized using granulated blast-furnace slag (GGBFS), while HSCM incorporated silica fume (SF) to achieve comparable compressive strength. Specimens were exposed to short-term elevated temperatures (200–600 °C) at 10 °C/min, with dwell times of 10–30 min, followed by furnace cooling or water quenching. Residual mechanical properties (compressive strength, tensile strength, impact resistance), thermal insulation, mass loss, and microstructural evolution were systematically analyzed. Results revealed that HSAAM exhibited complete spalling resistance up to 600 °C, whereas HSCM suffered partial spalling at 400 °C and catastrophic failure under water cooling. After 10 min at 400 °C, HSAAM retained 66.8% compressive strength (52 MPa) and 82% tensile strength (1.66 MPa), while HSCM retained 102% compressive strength (77.5 MPa) but experienced 10% specimen failure. HSAAM demonstrated superior thermal insulation, with core temperatures 44% lower than HSCM at 400 °C. Microstructural analysis via SEM/EDS identified a nano-porous matrix in HSAAM, facilitating vapor release and mitigating internal pressure. These findings position alkali-activated slag mortars as a robust, fire-resilient alternative to conventional cementitious systems in high-temperature environments.

## Introduction

Protecting concrete structures from fire-induced damage is essential, as it significantly reduces potential losses in structural integrity, economic costs, and human safety. Consequently, prioritizing research efforts on enhancing fire safety measures is of paramount importance. Ordinary Portland cement (OPC)- based concrete remains the most extensively used construction material worldwide, owing to its favorable mechanical properties and availability^1–3^. Ordinary high-strength composites (OHSC) exhibit greater susceptibility to strength and durability deterioration when compared to normal-strength concrete (NSC). Furthermore, OHSC is more prone to explosive spalling, as reported in several studies^4,5^. Instances of explosive spalling in OHSC have been documented at temperatures as low as 200 °C^6^ and at 300 °C in other investigations^4,7^ during furnace heating. This phenomenon is characterized by the sudden and violent disintegration of surface layers in the heated OHSC, primarily attributed to its low permeability and dense microstructural characteristics^4,8^. In addition to these safety concerns, the large-scale production of OPC-based concrete poses significant environmental challenges. The manufacturing process of OPC results in substantial carbon dioxide emissions and the depletion of non-renewable natural resources. It is estimated that approximately one ton of carbon dioxide is emitted for every ton of OPC produced, contributing to nearly 5–7% of global CO_2_ emissions^9–13^. The dual challenge of OHSC’s vulnerability to explosive failure at elevated temperatures and the detrimental environmental impact of OPC production has driven researchers to explore the development of innovative high-strength concrete alternatives that are both eco-friendly and resistant to fire-induced explosive spalling. Alkali-activated composites (AAC) have garnered considerable attention due to their potential as zero-cement binders, offering a sustainable alternative to conventional cementitious materials. In AAC systems, aluminosilicate-rich industrial by-products—such as blast furnace slag, fly ash, and metakaolin—are employed as precursor materials, which react with alkaline activator solutions to form new binding phases^14–19^. The alkali activation process leads to the formation of three primary gel types. The first is a three-dimensional sodium-alumino silicate hydrate (N-A-S-H) gel, typically produced when low-calcium precursors like Class F fly ash are utilized^20–23^. The second is calcium-alumino silicate hydrate (C-A-S-H) gel, which forms from calcium-rich precursors such as granulated blast-furnace slag. When both low- and high-calcium precursors are combined, an intermixing of C-A-S-H and N-A-S-H gels occurs, resulting in the formation of sodium-calcium-alumino silicate hydrate (N-C-A-S-H) gel alongside N-A-S-H gel phases^24,25^. AACs are recognized for their superior mechanical performance, enhanced durability, thermal stability, resistance to alkali-silica reaction (ASR), and improved resistance to acid attack and fire exposure^26–30^. The exceptional resistance of alkali-activated composites (AAC) to explosive spalling at elevated temperatures is primarily attributed to their inherent nano-porosity within the three-dimensional gel structure. This nano-porous network facilitates the migration and evaporation of both physically bound water and chemically bonded hydroxyl groups (OH⁻) without compromising the integrity of the matrix^2,22,31,32^. Consequently, AAC systems generally exhibit superior thermal stability and high-temperature performance when compared to ordinary Portland cement (OPC)-based materials^33–37^. However, despite their enhanced fire resistance, notable reductions in the compressive strength of alkali-activated pastes, mortars, and concretes have been reported following exposure to temperatures of 400 °C, 600 °C, and 800 °C, particularly when specimens are subjected to ambient air cooling^38–44^. For example, Cai and Ye^45^ examined the deterioration mechanisms of ultra-high-performance geopolymer concrete (UHPGC) under high-temperature conditions. Their findings indicated that the loss of strength below 600 °C is primarily associated with the decomposition and degradation of the calcium-aluminum silicate hydrate (C-A-S-H) phase, accompanied by the development of thermal cracking, increased porosity, and coarsening of the pore structure.

While these findings provide valuable insights into the degradation mechanisms, the experimental conditions in existing studies remain limited in scope. Prior investigations into the thermal performance of high-strength alkali-activated concrete have predominantly employed long soaking times ranging from 1 h to 3 h and slow cooling regimes at ambient temperatures^38–45^. Therefore, the present study aims to address this critical research gap by systematically evaluating the influence of rapid cooling via water quenching on high-strength alkali-activated mortars (HSAAM) subjected to elevated temperatures under short-term thermal exposure. Only Shaikh^46^ has investigated the effect of water cooling on normal-strength fly ash-based concrete. His findings revealed that, at 200 °C, the indirect tensile strength exhibited a slight improvement regardless of the cooling method employed. In contrast, numerous studies have explored the impact of water cooling on the mechanical properties of ordinary high-strength composites (OHSC)^47–56^. Several researchers have reported that, at temperatures exceeding 200 °C, water cooling results in higher residual compressive strength compared to air cooling. This improvement is attributed to the densification of the microstructure, as the rehydration process fills fissures and voids with hydration products, thereby reducing porosity and enhancing residual strength^47,49–55^. Conversely, other studies have documented a decline in residual compressive strength following water cooling, attributing this to thermal shock and the volumetric expansion of rehydration products, which induce additional cracking and weaken the material’s strength^46,48,56^. Although extensive research has been conducted on the thermal performance of high-strength cement mortars (HSCM) under both rapid and slow cooling regimes, most of these studies have adopted prolonged exposure durations—typically between 1 and 3 h—at elevated temperatures. Such conditions, however, do not accurately reflect the transient nature of most real fire events, particularly in the context of modern fire suppression technologies, where structural elements are often subjected to brief thermal shocks followed by abrupt cooling. Despite its practical relevance, this short-term exposure scenario remains significantly underexplored in the current literature.

To address this overlooked aspect, the present study offers a systematic evaluation of both high-strength cement mortar (HSCM) and high-strength alkali-activated mortars (HSAAM), carefully designed to exhibit comparable 28-day compressive strengths, thereby ensuring a fair and balanced comparison between the two systems. Specimens were exposed to elevated temperatures of 200, 400, and 600 °C for brief durations of 10, 20, and 30 min, followed by two distinct cooling regimes: rapid water quenching and controlled furnace cooling. The research aims to provide a comprehensive understanding of how these variables affect key performance indicators, including residual compressive strength, splitting tensile strength, impact resistance, mass loss, thermal insulation capacity, visual appearance, microstructural stability, and vulnerability to explosive spalling. This approach introduces a novel experimental framework that more realistically simulates emergency fire conditions and contributes valuable insights into the post-fire behavior of next-generation mortar systems.

## Experimental work

### Experimental program

**Fig. 1:**
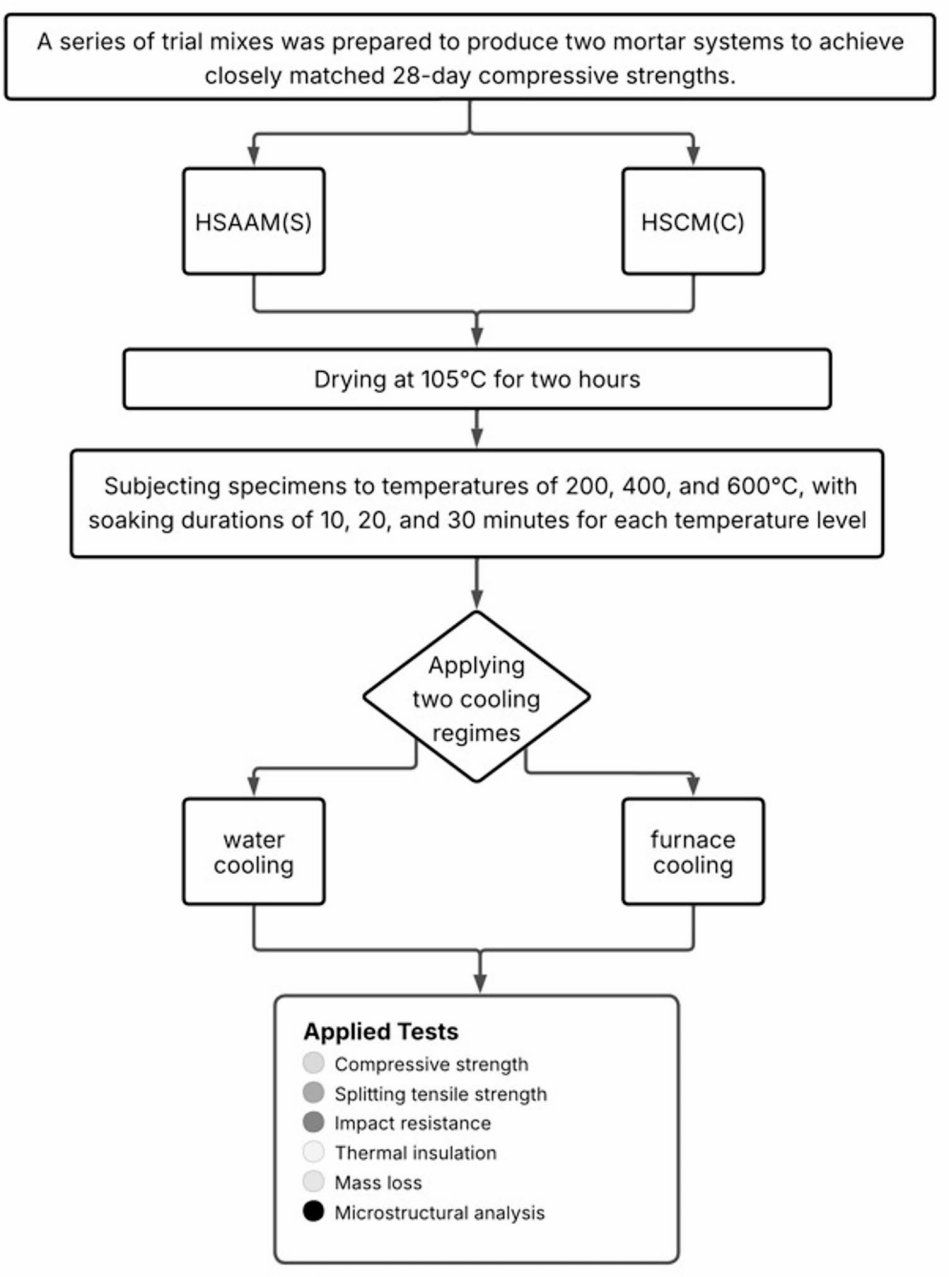
Schematic overview of the experimental program.

This study developed high-strength alkali-activated (HSAAM) and cement-based mortars (HSCM) with closely aligned 28-day compressive strengths to examine the effects of elevated temperatures (200, 400, and 600 °C), three soaking durations (10, 20, and 30 min), and two cooling regimes (furnace and water) on their performance, as shown in Fig. 1. A total of 36 distinct groups were produced to comprehensively evaluate these parameters. The compressive strength, splitting tensile strength, and impact resistance of the mixtures were assessed at both 7 and 28 days under ambient conditions, as well as after exposure to elevated temperatures at 28 days. For each testing condition, three identical specimens were utilized to ensure the reliability of the results. The detailed proportions of constituent materials required to produce 1 m³ of HSAAM and HSCM are provided in Tables 1 and 2. In this paper, the designation “S” refers to the HSAAM specimens, while “C” denotes the HSCM specimens. The numbers 10, 20, and 30 correspond to the soaking durations in minutes. Additionally, “F” and “W” are used to indicate furnace cooling and water cooling, respectively. For instance, the notation S/400/30/W identifies an HSAAM specimen subjected to 400 °C for 30 min, followed by water cooling.

**Table 1:** Constituent materials in kg required to produce 1 m^3^ from HSAAM.

Mix code	Slag (kg/m^3^)	Sand (kg/m^3^)	Na_2_SiO_3_ (kg/m^3^)	NaOH (kg/m^3^)	Water (kg/m^3^)
HSAAM	880	880	221.8	72.5	156

**Table 2:** Constituent materials in kg required to produce 1 m^3^ from HSCM.

Mix code	Cement	Silica fume	Sand	Water	Superplasticizer
HSCM	710	70	1354	203	23.4

### Materials characterization

**Fig. 2:**
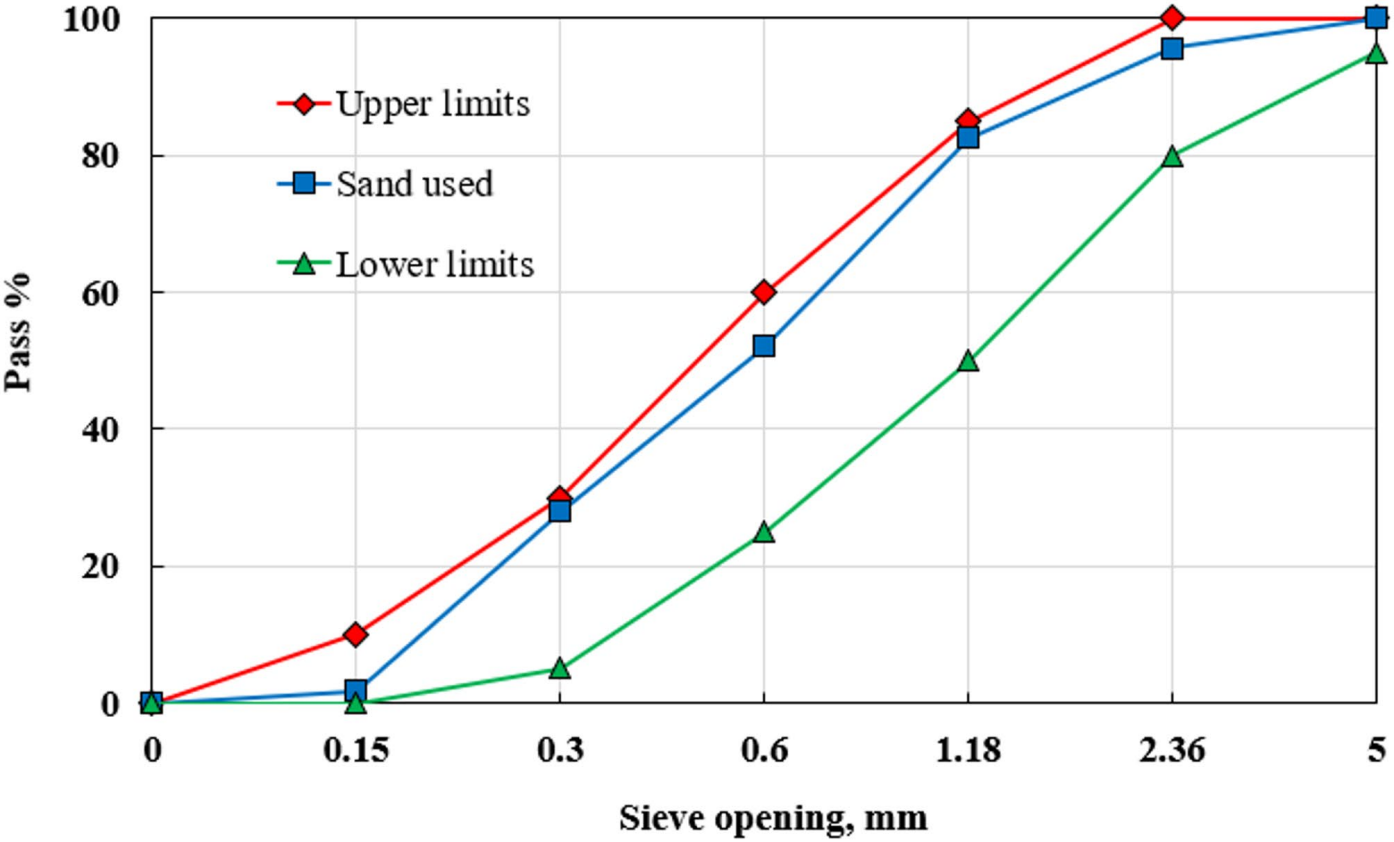
Grading curve of sand according to Egyptian standard specification limits^57^.

In this investigation, the high-strength alkali-activated mortar (HSAAM) was prepared using locally sourced ground granulated blast-furnace slag (GGBFS) as the primary precursor. The GGBFS, produced through rapid water cooling, possesses a specific gravity of 2.61. The chemical composition of the slag is presented in Table 3. The alkaline activator solution employed in synthesizing the HSAAM consisted of 99% pure sodium hydroxide (NaOH) pellets and a commercially available sodium silicate (Na_2_SiO₃) solution. The sodium silicate solution had a silica-to-sodium oxide (SiO_2_/Na_2_O) mass ratio of 2.58. The detailed chemical compositions of Na_2_SiO₃ and NaOH are provided in Tables 4 and 5, based on supplier specifications. For the production of high-strength cement mortar (HSCM), Ordinary Portland Cement (OPC) type CEM I 52.5 N was used as the primary binder, while silica fume (SF) with an average particle size of 7 μm served as a mineral admixture. The physical properties of both OPC and SF are also summarized in Table 3. Additionally, a superplasticizer was incorporated into the HSCM mixture at a dosage of 3% by weight of cement to enhance workability. Both mortar types utilized clean, natural sand with a particle size range of 0.15–5 mm as a fine aggregate. The sand had a specific gravity of 2.65 and a fineness modulus of 2.25. The particle size distribution of the fine aggregates is illustrated in Fig. 2.

**Table 3:** Chemical properties of OPC, GGBFS and SF (according to the supplier).

Materials	Chemical properties												
	SiO_2_	AL_2_O_3_	Fe_2_O_3_	CaO	MgO	SO_3_	K_2_O	Na_2_O	TiO_2_	P2O_5_	MnO	Cl	L.O. I
OPC	21.2	4.67	5.05	64.73	1.5	2.05	0.22	0.3	–	–	–	–	2.6
GGBFS	38.25	7.64	0.53	34.2	7.73	3.01	1.35	1.14	0.54	≥ 0.01	5.17	0.18	≤ 0.01
SF	88.9	0.29	0.94	0.2	0.36	5.12	–	0.52	–	–	–	–	3.4

**Table 4:** Chemical composition of sodium hydroxide.

Constituent	Amount
Na_2_O	60.25%
Water	39.75%

**Table 5:** Chemical composition of sodium silicate.

Constituent	Amount
Na_2_O	11.98%
SiO_2_	31%
Water	57%

### Mixing procedures

The mixing procedure for the high-strength alkali-activated mortar (HSAAM) was carried out in the following sequence: First, the alkaline activator solution was prepared by dissolving sodium hydroxide granules and adding sodium silicate solution to a pre-measured quantity of water, ensuring a consistent water-to-binder ratio of 0.35. The solution was stirred thoroughly until complete dissolution of the sodium hydroxide granules was achieved. Subsequently, the prepared alkaline solution was gradually added to the granulated blast-furnace slag (GGBFS) in a pan mixer and mixed for approximately 3 min. Following this, the fine aggregate (sand) was introduced, and the entire mixture was blended for an additional 5 min. For the high-strength cement mortar (HSCM), the mixing process involved initially dry-blending the measured quantities of ordinary Portland cement (OPC), silica fume (SF), and sand in a mechanical pan mixer for 2 min. Water and the superplasticizer were then gradually introduced, and mixing continued for an additional 5 min to ensure uniform consistency. Following mixing, the fresh mortar was cast into cubic molds and compacted using a vibrating table for 30 s to eliminate entrapped air.

### Heating and cooling procedure

**Fig. 3:**
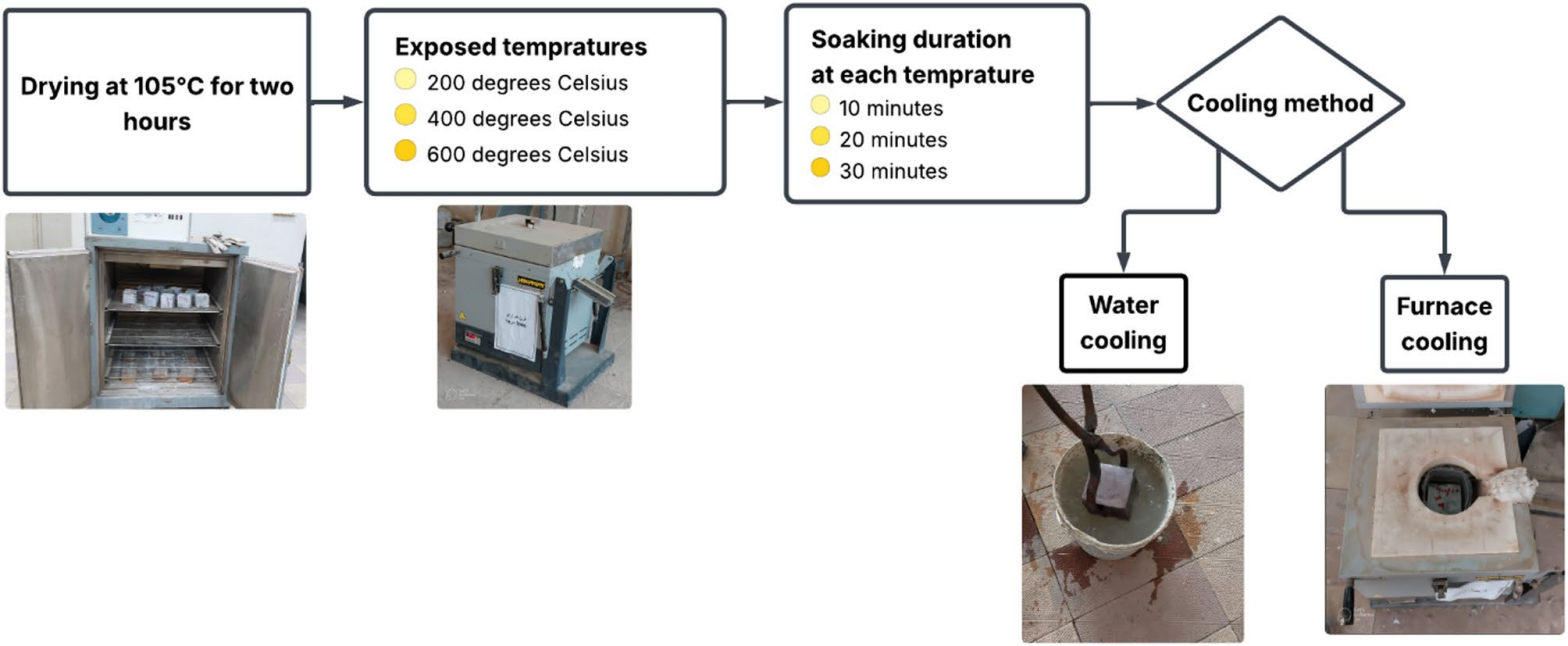
Schematic overview of heating and cooling procedure.

**Fig. 4:**
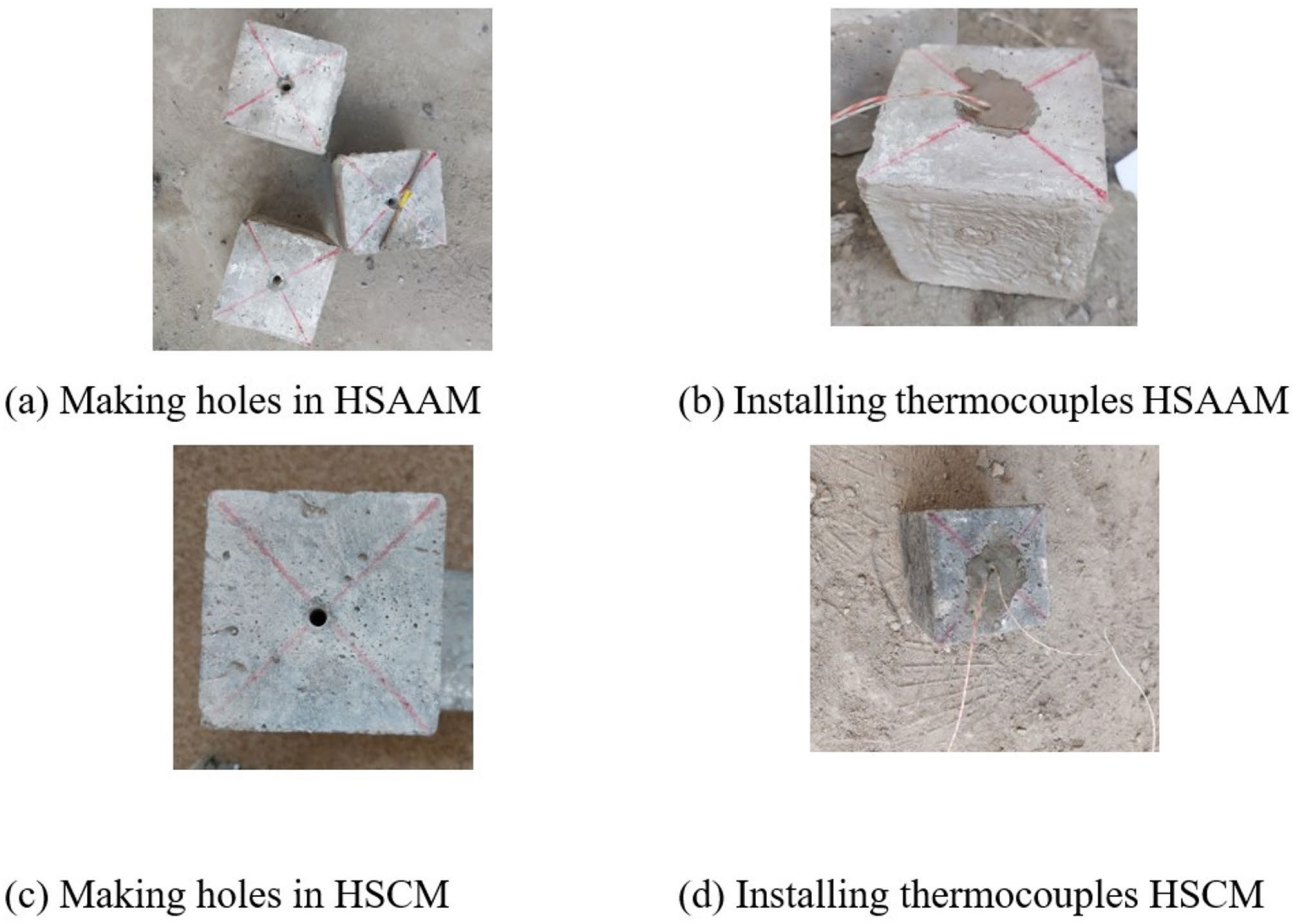
The procedures for installing type K thermocouples into cubes.

After 27 days of water curing, the specimens were demolded and left to dry under laboratory conditions for one day. Prior to the heating process, all specimens were oven-dried at 105 °C for two hours to minimize the risk of explosive spalling during thermal exposure. Subsequently, the specimens were subjected to a controlled heating regimen using a small electric furnace, as illustrated in Fig. 3. The furnace featured a heating chamber capable of accommodating two 100 mm × 100 mm × 100 mm cubes, with heating coils positioned on the vertical sides of the chamber. A uniform heating rate of 10 °C/min was applied, which is considered relatively high for larger specimens due to the associated risk of inducing thermal stresses, as noted in previous studies^47,48^. Once the target temperature (200, 400, or 600 °C) was reached, the specimens were held at that temperature for specified soaking durations of 10, 20, or 30 min. Following the heating phase, two distinct cooling regimes were applied. In the furnace cooling regime, specimens were left to cool gradually within the semi-open furnace until they returned to ambient temperature. For the water cooling regime, specimens were immediately removed from the furnace after the soaking period and immersed in room-temperature water for 5 min, after which they were allowed to cool naturally to room temperature. To monitor the internal temperature evolution and assess the thermal insulation behavior of both HSAAM and HSCM, type K thermocouples were embedded at the center of a sacrificial cube for each experimental condition, as shown in Fig. 4. These thermocouples were connected to a data logger, which recorded the core temperature at 5-second intervals throughout the heating and cooling processes. Given the limited dimensions of the furnace chamber, the furnace temperature closely approximated the surface temperature of the specimens. The installation procedure for the thermocouples involved drilling a 50 mm deep, 8 mm diameter hole at the center of each cube after 7 days of water curing. The thermocouple was inserted into the hole, which was subsequently filled with the same mortar used in the specimen. The modified specimens were then subjected to an additional 28 days of water curing to ensure complete integration.

### Compression, tension, impact, and microscopic analysis tests

Following exposure to elevated temperatures under varying soaking durations and subsequent cooling via furnace or water regimes, the specimens were left to cool for 24 h to ensure they reached ambient temperature prior to residual strength testing. Residual compressive strength was determined using a compression test on 100 mm × 100 mm × 100 mm cubes in accordance with BS EN 12390-3^58^. Residual splitting tensile strength was assessed following BS EN 12390-6^59^. Impact resistance was evaluated using the drop-weight method as specified in ACI 544.2R-89^60^. This test involves repeatedly releasing a 4.5 kg steel ball from a height of 450 mm, allowing it to fall freely under gravitational acceleration (9.81 m/s²). The number of blows required to initiate a visible hairline crack on the specimen’s upper surface (denoted as Ni) and to cause final failure (denoted as Nf), indicated by fragmentation or displacement of the specimen’s parts, was recorded. As a control reference, compressive strength, splitting tensile strength, and impact resistance were also evaluated at ambient temperature after 7 and 28 days. Microscopic analysis was carried out at the National Research Centre (Egypt) using a JEOL JSM-6510LV scanning electron microscope capable of magnifications up to 300,000×. Various magnification levels (1000×, 5000×, 10,000×, and 15,000×) were employed to closely examine the microstructural features of the specimens. Samples were extracted from fragments collected approximately 10 mm beneath the surface of crushed cubes post-28-day compressive testing. These samples were dried at 70 °C until a constant mass was achieved, then mounted on holders using carbon adhesive. A thin gold coating was applied via sputter coating to enhance conductivity and image resolution. Additionally, the elemental composition of the specimens was investigated using Oxford X-Max 20 energy-dispersive X-ray spectroscopy (EDS).

## Results and discussion

### Potential for explosion spalling and the visual appearance of C and S mixtures

**Fig. 5:**
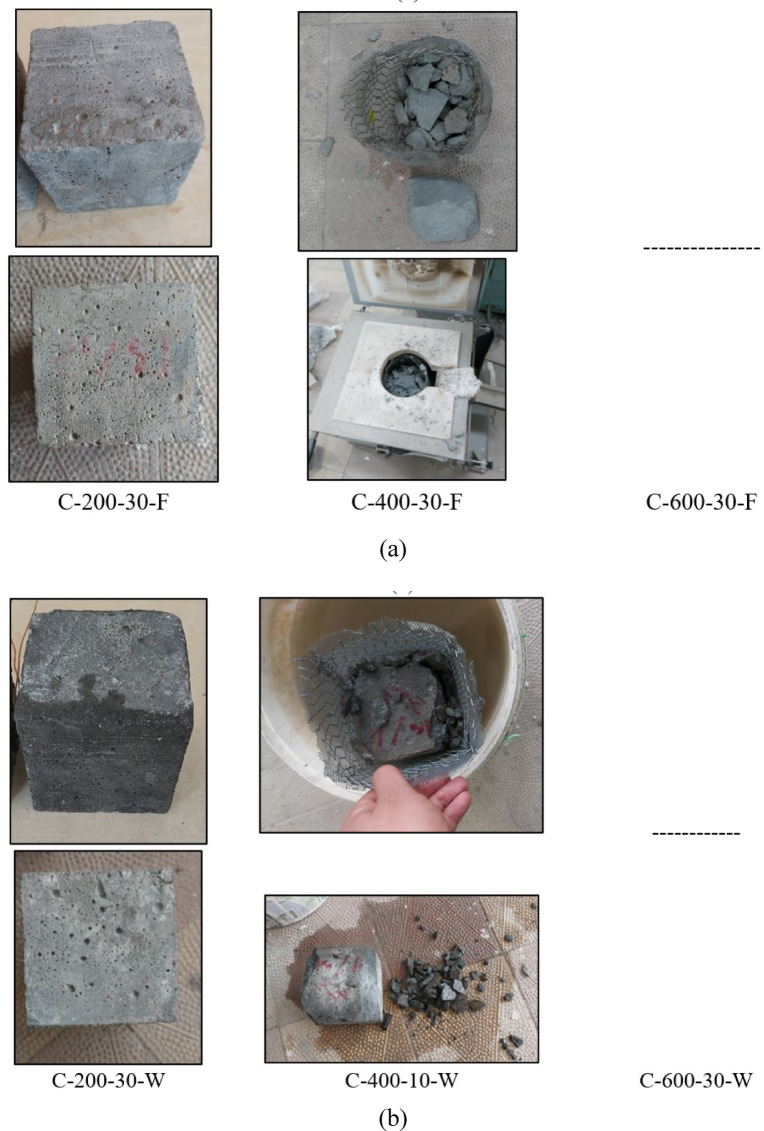
Visual appearance of C specimens (**a**) after furnace cooling, (**b**) after water cooling.

This section analyzes the effect of elevated temperatures and cooling methods (furnace and water cooling) at 200, 400, and 600 °C, with soaking durations of 10, 20, and 30 min, on the susceptibility of C and S specimens to explosive spalling. Spalling is defined as the sudden and violent detachment of a surface layer of concrete when exposed to high temperatures^7^. The results demonstrated that C specimens exhibited no signs of explosive spalling up to 400 °C when cooled in the furnace, i.e., no fragmentation was observed even after a soaking duration of 30 min. However, at 400 °C, when the heating duration ranged between 27 and 30 min, approximately 10% of the specimens experienced sudden and violent spalling during furnace exposure; see Fig. 5a. This spalling led to the fragmentation of the specimens into pieces ranging from 2 cm to 6.5 cm in length and 1.5 cm to 5 cm in thickness, leaving only about 35% of the original specimen volume intact. The occurrence of explosive spalling is primarily attributed to the dense microstructure of the material, which impedes the dissipation of internal pressure caused by the evaporation of physically and chemically bonded water. Additionally, a substantial thermal gradient of 181.3 °C between the specimen’s surface and core induced significant thermal stresses. The combined effects of increased internal vapor pressure and thermal stresses ultimately led to the violent failure of the specimens.

Regarding water cooling, C specimens exhibited no signs of explosive spalling up to 400 °C at all heating durations when exposed to 200 °C, see Fig. 5a and b. At 400 °C, all specimens heated for 10, 20, and 30 min and subsequently immersed in water experienced explosive spalling, as illustrated in Fig. 5b. When the specimen was heated for 10 min, the fragmentation resulting from the spalling event produced debris ranging from 1 cm to 3.5 cm in length and 0.5 cm to 2.5 cm in thickness, leaving approximately 70% of the original specimen volume intact, as illustrated in Fig. 5b. This failure can be attributed to the thermal shock induced by sudden immersion, which generated excessive thermal stresses surpassing the mortar’s tensile strength, in addition to the internal vapor pressure buildup. As a result, reliable data for water-cooled specimens at 400 °C could not be obtained. Moreover, at 600 °C, no results could be recorded as all C specimens underwent explosive spalling inside the furnace before reaching the target temperature.

**Fig. 6:**
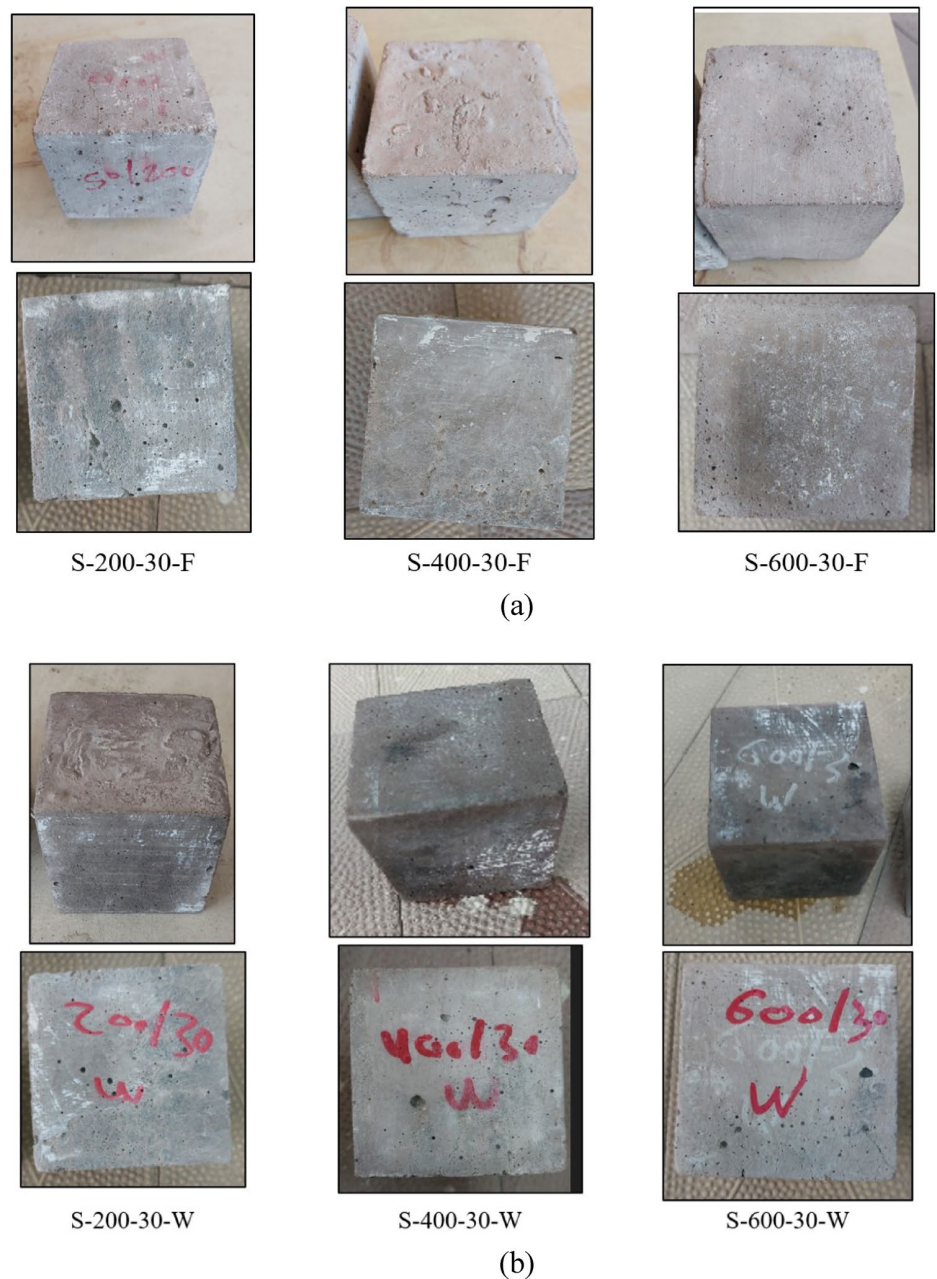
Visual appearance of S specimens (**a**) after furnace cooling, (**b**) after water cooling.

Conversely, the alkali-activated mixture exhibited superior resistance to explosive spalling. The results indicated that S specimens withstood exposure up to 600 °C at all soaking durations (10, 20, and 30 min), irrespective of the cooling method. Unlike the C specimens, i.e., no fragmentation was observed, see Fig. 6a, b. This exceptional resistance can be attributed to the intrinsic nano-porosity of the alkali-activated matrix, which facilitates the escape and evaporation of physically and chemically bonded water (hydroxyl groups, OH)^2,22,31,32^. Furthermore, the pre-existing microcracks in the alkali-activated matrix, resulting from the rapid reaction at elevated temperatures during mixing and the high alkalinity, likely enhance the void network connectivity. This improved network allows for efficient vapor release, thereby preventing the buildup of internal pressure and eliminating explosive spalling at high temperatures^61–63^.

### Effect of soaking times and cooling regimes on the compressive strength of the S mixture

**Fig. 7:**
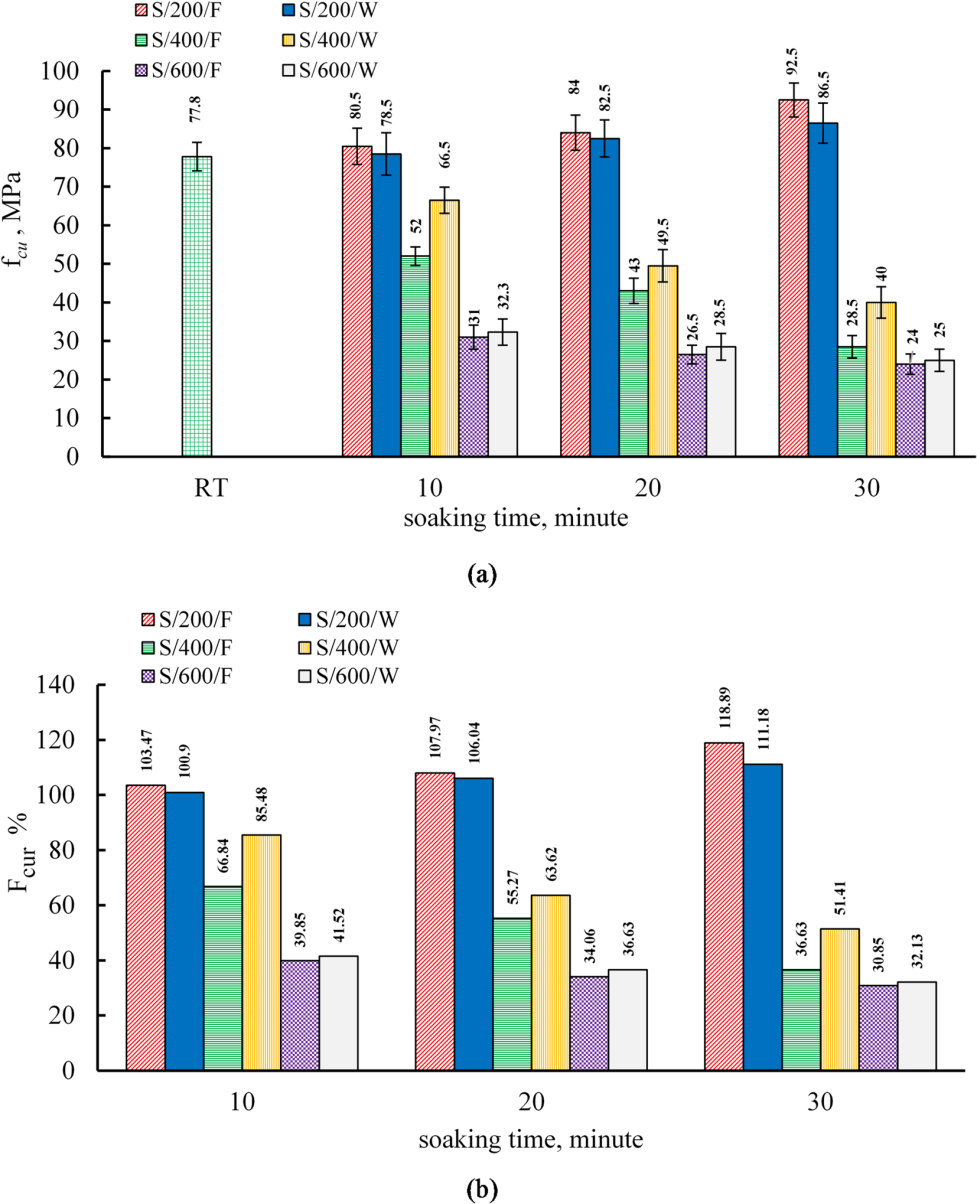
(**a**) The results of compressive strength and (**b**) relative residual compressive strength of S specimens.

The influence of elevated temperatures (200, 400, and 600 °C) with soaking durations of 10, 20, and 30 min, along with two cooling regimes (furnace and water cooling), on the residual compressive strength of S mix cube specimens was investigated, as illustrated in Fig. 7. The average residual compressive strength values before and after heating are presented in Table 6. At ambient conditions, the compressive strength of the S mix was measured at 70 MPa and 77.8 MPa at 7 and 28 days, respectively.

As shown in Fig. 7, at 200 °C, the residual compressive strength exhibited an increase relative to its room-temperature value. In furnace-cooled specimens, the compressive strength increased by 3.47%, 7.97%, and 18.89% for soaking times of 10, 20, and 30 min, respectively. Conversely, water-cooled specimens exhibited lower increases of 0.9%, 6.04%, and 11.18% for the same soaking durations. These findings indicate that longer exposure to heat enhances compressive strength, regardless of the cooling method, though furnace cooling resulted in a greater strength improvement than water cooling. This trend is consistent with previous studies that reported similar behavior at 200 °C^37,52,64^. The increase in compressive strength at this temperature can be attributed to the densification and stiffening of N-A-S-H and C-A-S-H gels due to heat exposure^37,64,65^. Additionally, some researchers have suggested that this strength enhancement may be linked to the additional hydration of unhydrated binder particles, facilitated by steam effects under what is known as the internal autoclaving effect^52,66^. The superior strength gain observed in furnace-cooled specimens compared to water-cooled ones may be explained by the rapid cooling effect of water, which potentially reduces the extent of beneficial heat-induced rehydration of anhydrous phases. At 200 °C, the highest recorded compressive strength was 92.5 MPa for furnace-cooled specimens subjected to a 30-minute soaking period.

At 400 °C, the compressive strength exhibited a significant reduction compared to room-temperature values. For furnace-cooled specimens, the strength decreased by 33.16%, 44.73%, and 63.37% at soaking times of 10, 20, and 30 min, respectively. In contrast, water-cooled specimens showed a relatively lower reduction of 14.52%, 36.38%, and 48.59% for the same soaking durations. These results indicate that prolonged exposure to elevated temperatures negatively affects residual compressive strength, with furnace-cooled specimens exhibiting greater strength loss than their water-cooled counterparts.

This trend is consistent with findings reported by Koskal et al.^55^, which demonstrated that water cooling preserves higher residual compressive strength compared to air cooling. A plausible explanation is the rehydration of thermally degraded phases, which contributes to filling microcracks and voids, thereby improving the material’s structural integrity^47,49–55^. Moreover, rapid cooling in water reduces prolonged exposure to high temperatures, limiting microstructural coarsening and subsequent strength degradation^67–69^. Regardless of the cooling method, the decline in compressive strength at 400 °C can be attributed to dehydration-induced shrinkage and the decomposition of calcium-aluminum silicate hydrate (C-A-S-H) phases. These factors, combined with thermal cracking, increased porosity, and coarsening of the pore structure, significantly impair mechanical performance^37,45,46,61,70^.

At 600 °C, the residual compressive strength exhibited a significant decline compared to its room-temperature value. For furnace-cooled specimens, the strength decreased by 60.15%, 65.94%, and 69.15% at soaking times of 10, 20, and 30 min, respectively. Similarly, water-cooled specimens experienced reductions of 58.48%, 63.37%, and 67.87% for the corresponding soaking durations. These results indicate that furnace-cooled specimens exhibit slightly greater losses than their water-cooled counterparts, consistent with the trend observed at 400 °C.

The pronounced strength reduction at 600 °C is primarily attributed to the extensive formation of microcracks and increased porosity, resulting from the decomposition of reaction products and the crystallization of phases such as eckermannite, anorthite, and nepheline^37,45,46,61,70^. Additionally, the thermal expansion of quartz in sand at temperatures exceeding 573 °C exacerbates structural damage. The phase transition from alpha-quartz to beta-quartz induces an approximate volumetric expansion of 0.85%, contributing to further microcracking and weakening of the matrix^48,71^. Moreover, the difference in residual strength between water- and furnace-cooled specimens at 600 °C was relatively small. This suggests that the majority of microstructural damage, including crack formation and pore coarsening, occurs at high temperatures (≥ 600 °C), reducing the influence of the cooling method on residual strength^72^.

**Table 6:** The average compressive strength results for the S mixture at room temperature (RT) and after furnace and water cooling.

Mixture	Compressive strength after furnace cooling (MPa)										
	RT		200 °C			400 °C			600 °C		
	7	28	10	20	30	10	20	30	10	20	30
S	70.0 (0.060) *	77.8 (0.048) *	80.5 (0.058) *	84.0 (0.054) *	92.5 (0.048) *	52.0 (0.046) *	43.0 (0.077) *	28.5 (0.101) *	31.0 (0.100) *	26.5 (0.091) *	24.0 (0.110) *
			Compressive strength after water cooling (MPa)								
			78.5 (0.070)	82.5 (0.058)	86.5 (0.060)	66.5 (0.051)	49.5 (0.085)	40.0 (0.103)	32.3 (0.105)	28.5 (0.121)	25.0 (0.115)

*Values in the parentheses indicate the coefficient of variance (dimensionless).

### Effect of soaking times and cooling regimes on the compressive strength of the C mixture

**Fig. 8:**
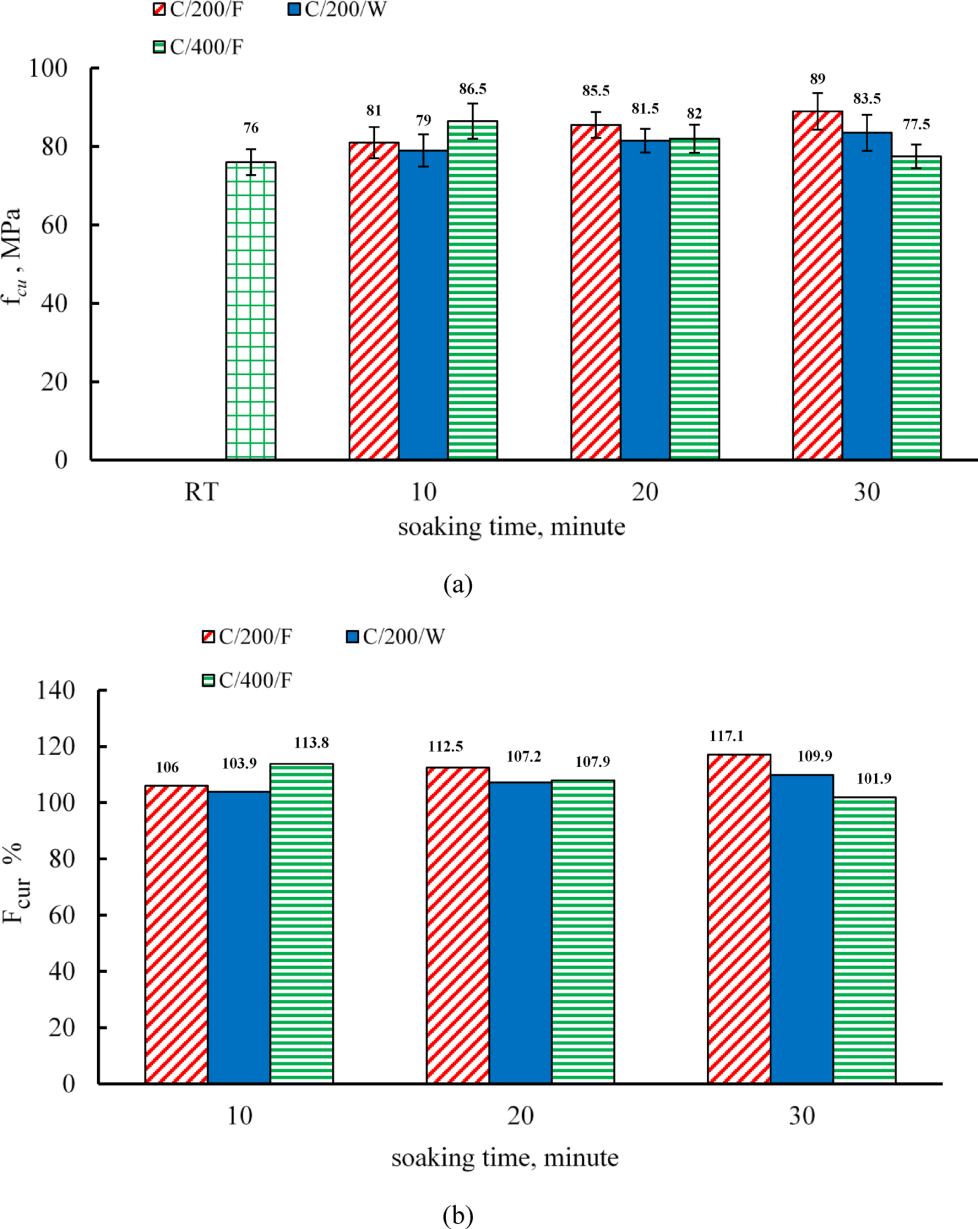
(**a**) The compressive strength for C, (**b**) relative residual compressive strength for C.

The residual compressive strength values of the C mix before and after heating to different temperatures are summarized in Table 7. The compressive strength was recorded at room temperature as 61 MPa at 7 days and 76 MPa at 28 days. As shown in Fig. 8, at 200 °C, furnace-cooled specimens exhibited compressive strength gains of 6.6%, 12.5%, and 17.1% at soaking durations of 10, 20, and 30 min, respectively, whereas water-cooled specimens achieved smaller increases of 3.9%, 7.2%, and 9.9% for the same durations. These findings confirm that prolonged exposure at 200 °C enhances residual compressive strength for both cooling methods, with furnace-cooled specimens achieving greater gains—a trend consistent with the results reported by Kara and Arslan^52^. This improvement is mainly linked to thermally induced secondary hydration and pozzolanic reactions, forming additional C–S–H gels^52,73,74^. The lower gain in water-cooled specimens may result from rapid cooling, which restricts these processes. At 200 °C, the highest compressive strength observed was 89 MPa for furnace-cooled specimens after 30 min of soaking.

As depicted in Fig. 8, at 400 °C, the compressive strength of furnace-cooled specimens increased by 13.8%, 7.9%, and 1.9% for soaking times of 10, 20, and 30 min, respectively, compared to the room-temperature strength. These results suggest that while heating initially enhances compressive strength, the extent of this increase diminishes as the soaking duration extends. The highest compressive strength at this temperature was recorded at 86.5 MPa for a soaking time of 10 min. The improvement in compressive strength at 400 °C can be attributed to the increased stiffness of the cement gel and the enhancement of surface forces between gel particles due to the release of adsorbed moisture^6,75^. Djaknoun et al.^76^ observed similar behavior in high-strength mortar, reporting an increase in mechanical properties up to 300 °C when cooled in air after being heated for two hours at a rate of 3.3 °C/min, followed by a decline at higher temperatures. At 400 °C, this strength enhancement has been attributed to additional hydrothermal reactions of unhydrated cement particles and the pozzolanic interaction of silica fume with calcium hydroxide^66,77–80^. However, the specific temperature at which compressive strength begins to decrease varies based on several factors, including the aggregate-to-binder ratio, heating rate, water-to-binder ratio, and specimen geometry^6^.

At 400 °C, prolonged exposure resulted in a progressive reduction in residual compressive strength, consistent with the observations reported by Culfik and Ozturan^81^. This decline can be attributed to the coarsening of the pore structure at elevated temperatures, as the decomposition of hydrated compounds increases the average pore radius, thereby weakening the material strength^67–69^. Additionally, previous studies have established an inverse relationship between the compressive strength of hardened cement paste and its pore volume^82,83^. Furthermore, even in the absence of significant decomposition of its constituents, concrete may experience a deterioration in its residual mechanical properties. This degradation is primarily caused by the combined effects of high temperature, vapor pressure buildup, and thermal stresses^84–86^.

**Table 7:** The average compressive strength results for the C mixture at room temperature (RT) and after furnace and water cooling.

Mixture	Compressive strength after furnace cooling (MPa)										
	RT		200 °C			400 °C			600 °C		
	7	28	10	20	30	10	20	30	10	20	30
**C**	61.0 (0.054)*	76.0 (0.043)*	81.0 (0.049)*	85.5 (0.038)*	89.0 (0.053)*	86.5 (0.052)*	82.0 (0.044)*	77.5 (0.038)*	–	–	–
			Compressive strength after water cooling (MPa)								
			79.0 (0.051)	81.5 (0.037)	83.5 (0.055)	–	–	–	–	–	–

*Values in the parentheses indicate the coefficient of variance (dimensionless).

**Effect of soaking time and cooling regimes on the splitting tensile strength of the S mixture**.

**Fig. 9:**
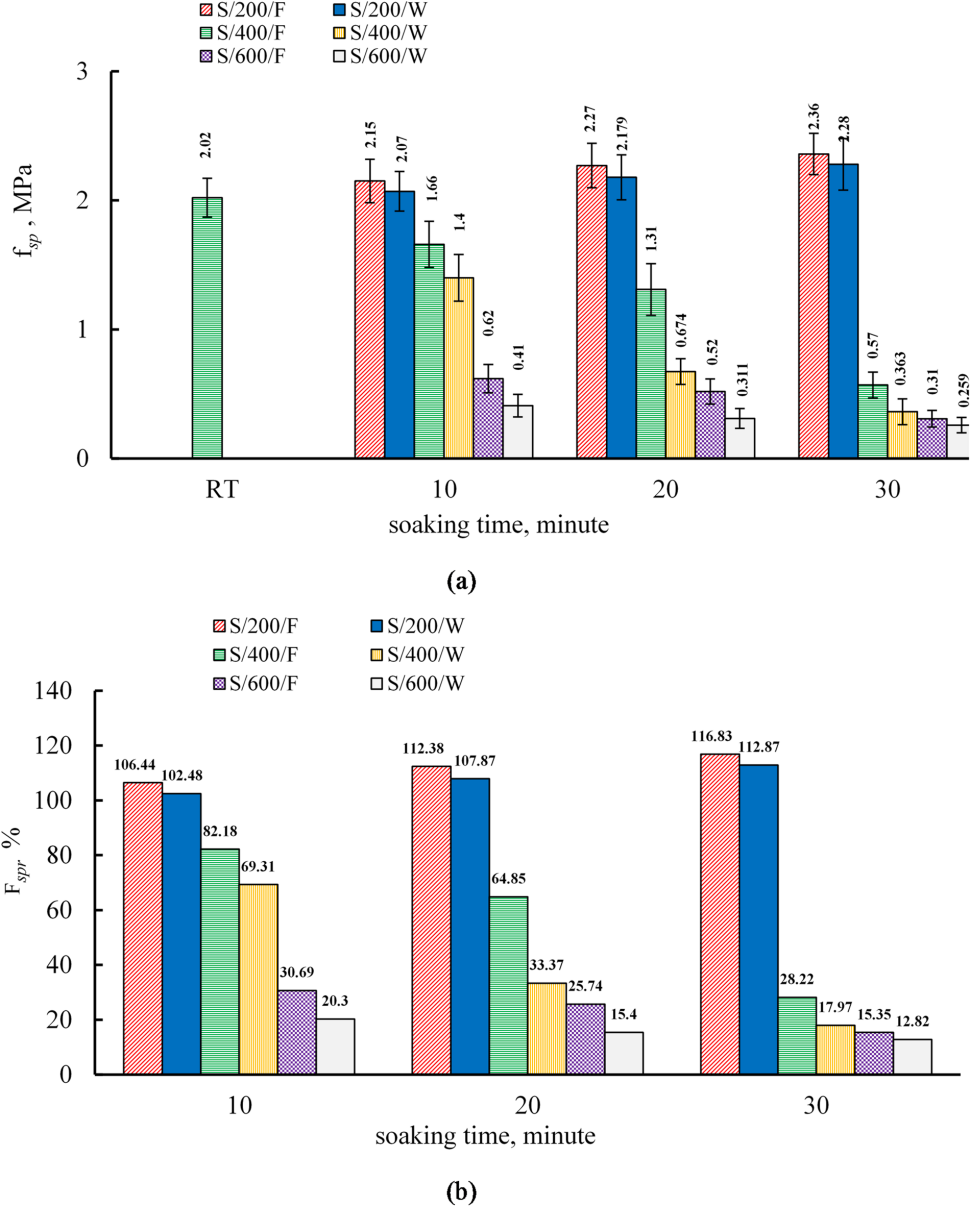
(**a**) The results of splitting strength and (**b**) relative residual splitting tensile strength of S specimens.

The following analysis elucidates the residual splitting tensile strength variance for S specimens subjected to different cooling methods, namely water cooling and furnace cooling, as depicted in Fig. 9. At ambient temperature, the splitting tensile strength values were 1.65 MPa and 2.02 MPa at 7 and 28 days, respectively, as documented in Table 8. These tensile strength values correspond to 2.6% and 2.35% of the compressive strength values at room temperature at 7 and 28 days, respectively. The relatively low tensile strength can be attributed to the formation of microcracks, which result from the reactions occurring at elevated temperatures and high alkalinity, as evidenced by the SEM images^61–63^. At 200 °C, the splitting tensile strength followed the same enhancement trend as the compressive strength. For furnace-cooled specimens, it increased by 6.44%, 12.38%, and 16.83% at soaking times of 10, 20, and 30 min, respectively, while for water-cooled specimens, the corresponding increases were 2.48%, 7.87%, and 12.87%. These gains, more pronounced in furnace-cooled specimens, arise from the same mechanisms previously discussed for compressive strength at this temperature. Up to 550 °C, fine aggregates remain largely unaffected by temperature, unlike coarse aggregates, with significant sand expansion occurring only at 573 °C^71,87^Accordingly, in mix C, the splitting tensile strength increased at 200 °C despite the presence of sand, contrary to mixtures containing both coarse and fine aggregates, where tensile strength decreases at all temperatures^46^. At 400 °C, the splitting tensile strength decreased markedly compared to room temperature. For furnace-cooled specimens, the reductions were 17.82%, 35.15%, and 71.78% at soaking times of 10, 20, and 30 min, respectively, while water-cooled specimens showed greater losses of 30.69%, 66.36%, and 82.03%. The decline intensified with soaking time for both cooling methods, with water cooling causing more severe losses than furnace cooling. This behavior is attributed to thermal shock from rapid water cooling, which generates internal stresses and microcracks^46,56^. Similarly, Koskal et al.^55^ found greater tensile strength loss in water-cooled specimens due to increased brittleness.

At 600 °C, the splitting tensile strength decreased by 69.31%, 74.26%, and 84.65% for furnace-cooled specimens, and by 74.7%, 84.6%, and 87.8% for water-cooled specimens at soaking times of 10, 20, and 30 min, respectively. Losses increased with soaking time, with slightly higher reductions under water cooling. This severe deterioration is linked to increased porosity from phase decomposition and the formation of crystalline phases such as eckermannite, anorthite, and nepheline, along with sand expansion above 573 °C^37,45,46,61,70^. The small difference between cooling methods indicates comparable mechanical degradation at this temperature.

**Table 8:** The average results of splitting tensile strength for the S mixture at room temperature (RT) and after furnace and water cooling.

Mixture	Splitting tensile strength after furnace cooling (MPa)										
	RT		200 °C			400 °C			600 °C		
	7	28	10	20	30	10	20	30	10	20	30
**S**	1.65 (0.092) *	2.02 (0.074) *	2.15 (0.078) *	2.27 (0.0.076) *	2.36 (0.068) *	1.66 (0.107) *	1.31 (0.153) *	0.57 (0.175) *	0.62 (0.177) *	0.52 (0.188) *	0.31 (0.209) *
			Splitting strength after water cooling (MPa)								
			2.07 (0.074)	2.18 (0.080)	2.28 (0.088)	1.40 (0.129)	0.67 (0.147)	0.36 (0.275)	0.41 (0.212)	0.31 (0.248)	0.26 (0.232)

*Values in the parentheses indicate the coefficient of variance (dimensionless).

### Effect of soaking time and cooling regimes on the splitting tensile strength of C mixture

**Fig. 10:**
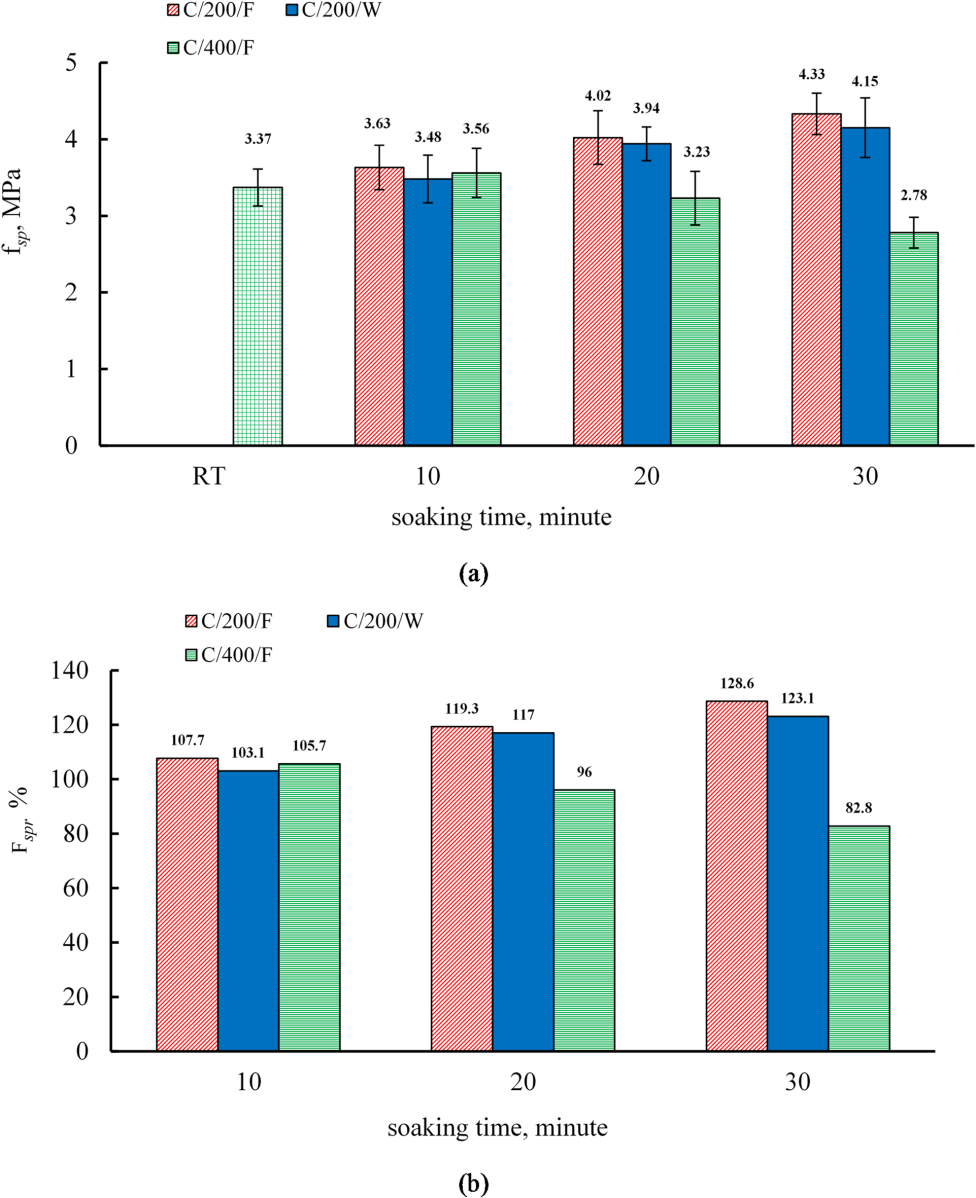
(**a**) The results of splitting strength and (**b**) relative residual splitting tensile strength of C specimens.

Figure 10 illustrates the variance in residual splitting tensile strength for C specimens that were cooled either in water or in a furnace. At room temperature, the splitting tensile strength values were 2.56 MPa and 3.37 MPa at 7 and 28 days, respectively, as documented in Table 9. At 200 °C, the splitting tensile strength increased progressively with soaking time, reaching 7.7%, 19.3%, and 28.6% at 10, 20, and 30 min, respectively, for furnace-cooled specimens, and 3.1%, 17%, and 23.1% for water-cooled specimens, with greater gains under furnace cooling.

At 400 °C, the splitting tensile strength in mix C was more adversely affected than the compressive strength. Furnace-cooled specimens gained compressive strength at all soaking times (10, 20, and 30 min), whereas tensile strength increased only by 5.7% at 10 min and decreased by 4% and 17% at 20 and 30 min, respectively. This discrepancy can be attributed to the fact that splitting tensile strength is more sensitive to microcracks and voids generated due to the decomposition of hydrated phases at high temperatures than compressive strength^6,61^.

**Table 9:** The average results of splitting tensile strength for the C mixture at room temperature (RT) and after furnace and water cooling.

Mixture	Splitting tensile strength after furnace cooling (MPa)										
	RT		200 °C			400 °C			600 °C		
	7	28	10	20	30	10	20	30	10	20	30
**C**	2.56 (0.077) *	3.37 (0.071) *	3.63 (0.079) *	4.02 (0.087) *	4.33 (0.062) *	3.56 (0.090) *	3.23 (0.108) *	2.78 (0.072) *	–	–	–
			Splitting tensile strength after water cooling (MPa)								
			3.48 (0.089)	3.94 (0.056)	4.15 (0.094)	–	–	–	–	–	–

*Values in the parentheses indicate the coefficient of variance (dimensionless).

### Effect of soaking time and cooling regimes on the impact energy of the S mixture

**Fig. 11:**
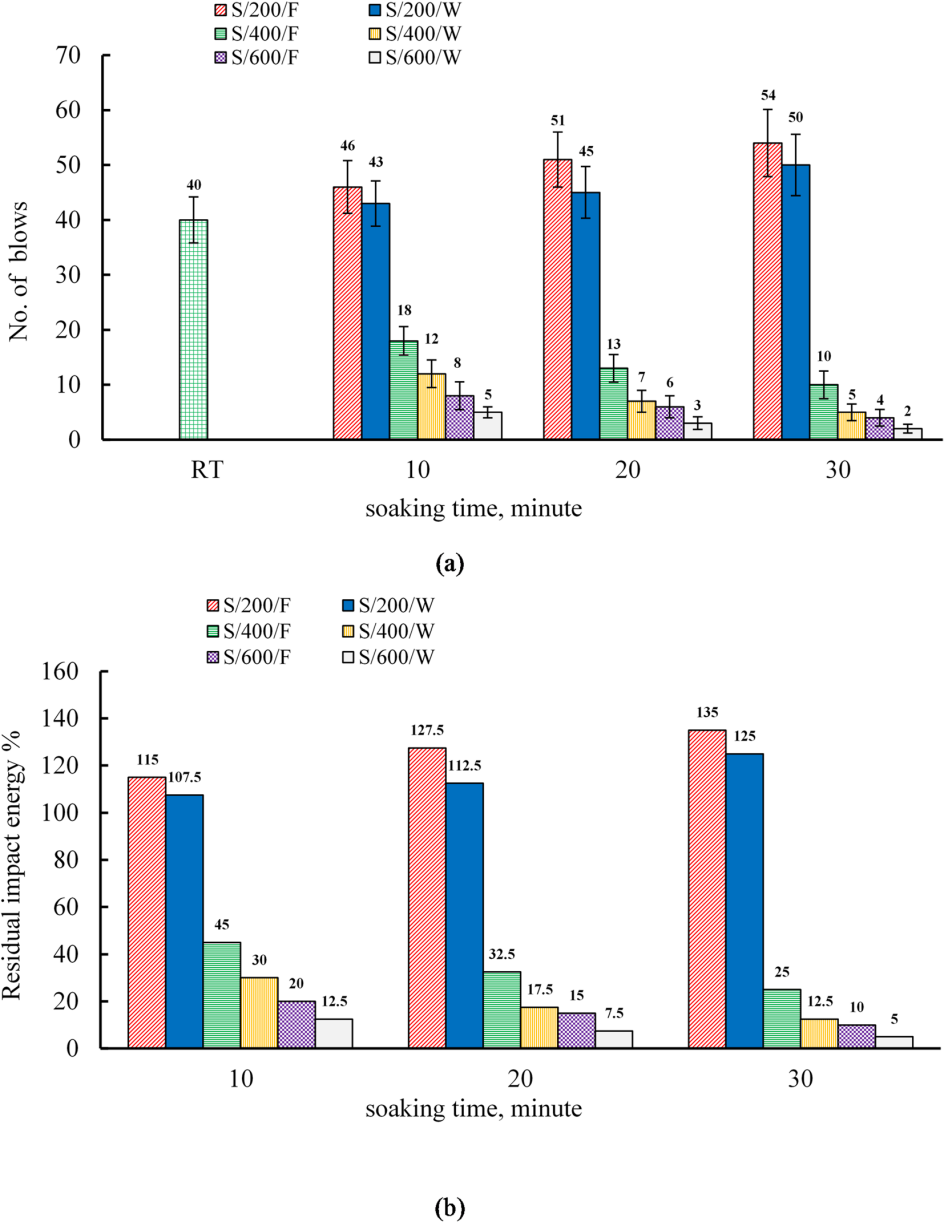
(**a**) The results of impact resistance and (**b**) the relative residual impact resistance of S specimens.

The drop weight impact test results for the two mixtures (S and C) manufactured in this research revealed that all specimens exhibited only a first fracture (N_i_), with no final fracture occurring. This can be attributed to the high strength of the base material, which results in increased brittleness and consequently weakens the material’s resistance to crack propagation^88,89^. As a result, once a crack initiates, a fracture occurs. Therefore, the impact energy of the specimens was calculated based on N_i_=N_f_. Figure 11 illustrates the variance in impact energy for S specimens that were cooled in water and a furnace at soaking times of 10, 20, and 30 min. At room temperature, the impact energy values were 650.56 kN·mm and 813.2 kN·mm at 7 and 28 days, respectively, as shown in Table 10. At 200 °C, the impact energy of furnace-cooled specimens increased by 15.0%, 27.5%, and 35.0% at soaking durations of 10, 20, and 30 min, respectively, while water-cooled specimens showed smaller gains of 7.5%, 12.5%, and 25.0%. The enhancement was more pronounced under furnace cooling, consistent with trends for compressive and splitting tensile strengths.

At 400 °C, furnace-cooled specimens exhibited impact energy reductions of 55.0%, 67.5%, and 75.0% at 10, 20, and 30 min, whereas water-cooled specimens experienced greater declines of 70.0%, 82.5%, and 87.5%. The more severe degradation under water cooling parallels its higher loss in splitting tensile strength at the same temperature. At 600 °C, impact resistance decreased further, with furnace-cooled specimens showing losses of 80.0%, 85.0%, and 90.0%, and water-cooled specimens 87.5%, 92.9%, and 95.0% at 10, 20, and 30 min, respectively. These substantial reductions, similar for both cooling regimes, correspond to the severe deterioration observed in splitting and compressive strengths for mixture S.

**Table 10:** The average impact energy results for the S mixture at room temperature (RT) and after furnace and water cooling.

Mixture	Impact energy after furnace cooling. (kN mm)										
	RT		200 °C			400 °C			600 °C		
	7	28	10	20	30	10	20	30	10	20	30
**S**	650.6 (0.097)*	813.2 (0.105) *	935.2 (0.104) *	1036.9 (0.098) *	1097.8 (0.113) *	366.0 (0.114) *	264.3 (0.193) *	203.3 (0.251) *	162.6 (0.314) *	122.0 (0.333) *	81.3 (0.375) *
			Impact energy after water cooling. (kN mm)								
			874.2 (0.096)	914.9 (0.104)	1016.5 (0.111)	244.0 (0.209)	142.3 (0.286)	101.7 (0.304)	101.7 (0.200)	61.0 (0.383)	40.7 (0.400)

*Values in the parentheses indicate the coefficient of variance (dimensionless).

### Effect of soaking time and cooling regimes on the impact energy of the C mixture

**Fig. 12:**
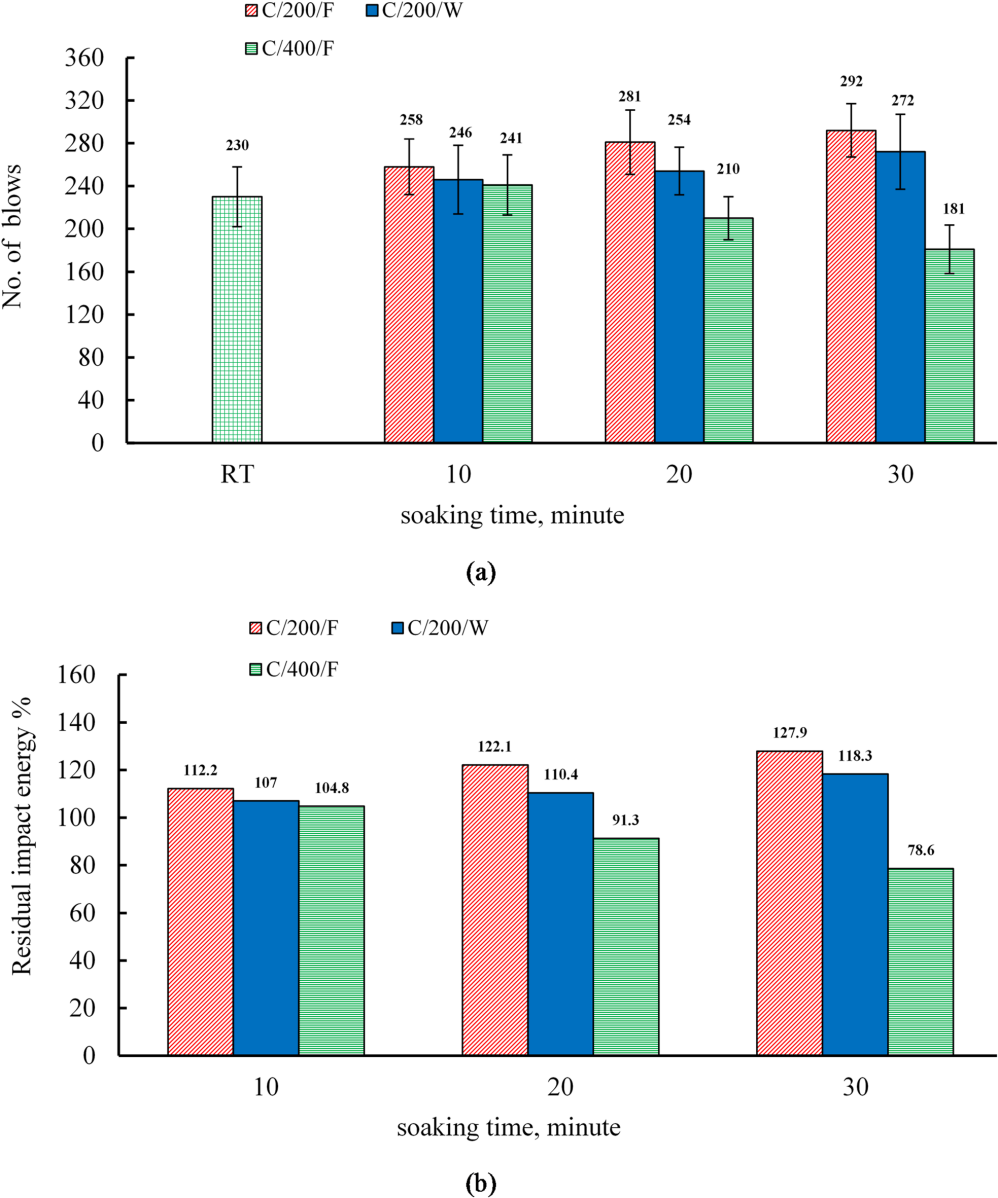
(**a**) The results of impact resistance and (**b**) the relative residual impact resistance of C specimens.

Figure 12 presents the variation in impact energy for C specimens that were subjected to different cooling methods (water and furnace) at various soaking times (10, 20, and 30 min). At ambient temperature, the impact energy values were 2764.88 kN · mm and 4675.9 kN · mm at 7 and 28 days, respectively, as detailed in Table 11. At 200 °C, the impact energy of furnace-cooled specimens increased by 12.2%, 22.2%, and 27.0% at soaking durations of 10, 20, and 30 min, respectively, whereas water-cooled specimens exhibited smaller gains of 7.0%, 10.4%, and 18.3%. The enhancement was consistently higher for furnace-cooled specimens, aligning with trends in compressive and splitting tensile strengths.

At 400 °C, impact resistance showed greater thermal sensitivity than compressive strength. A slight gain of 4.8% was recorded after 10 min, while longer exposures of 20 and 30 min resulted in declines of 8.7% and 21.4%, respectively. These results confirm that prolonged exposure at elevated temperatures leads to progressive reductions in residual impact energy.

**Table 11:** The average impact energy results for the C mixture at room temperature (RT) and after furnace and water cooling.

Mixture	Impact energy after furnace cooling. (kN·mm)										
	RT		200 °C			400 °C			600 °C		
	7	28	10	20	30	10	20	30	10	20	30
**C**	2764.9 (0.136)*	4675.9 (0.122)*	5245.1 (0.100)*	5712.7 (0.107)*	5936.4 (0.087)*	4899.5 (0.116)*	4269.3 (0.095)*	3679.7 (0.124)*	–	–	–
			Impact energy after water cooling. (kN·mm)								
			5001.2 (0.130)	5163.8 (0.087)	5529.8 (0.129)	–	–	–	–	–	–

*Values in the parentheses indicate the coefficient of variance (dimensionless).

### The effect of binder type on thermal insulation properties

**Fig. 13:**
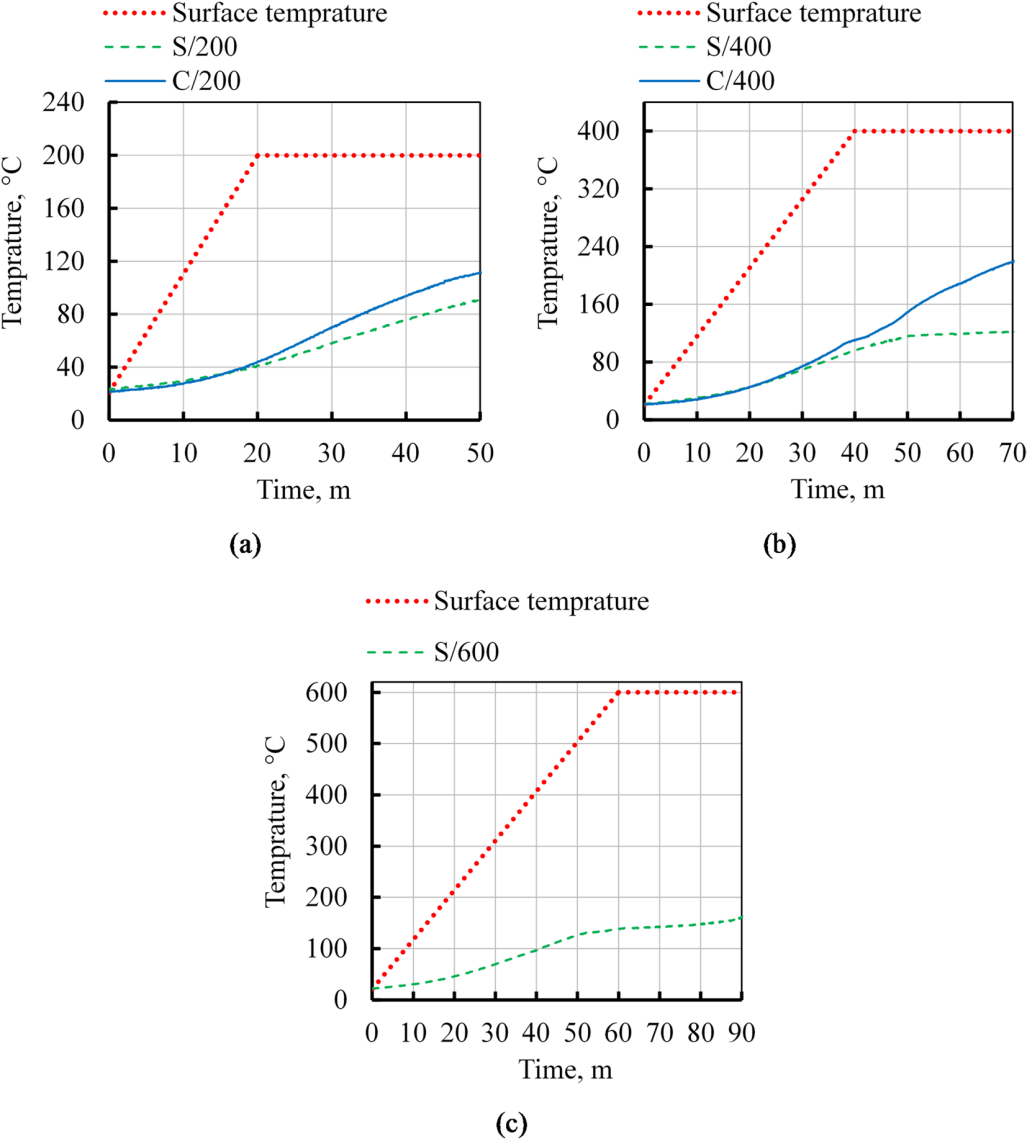
The time-temperature profiles for both the surface and core of the specimens of S and C at temperatures: (**a**) 200 °C, (**b**) 400 °C, and (**c**) 600 °C.

Figure 13 illustrates the comparative thermal insulation capabilities of mixtures S and C, contingent upon the type of binder utilized. The findings indicate that at temperatures of 200 and 400 °C, the slag-based material demonstrated superior thermal insulation performance compared to the cement-based material. As depicted in Fig. 13, the disparity between the core temperatures of the two samples widens as the target temperature escalates. After a 30-minute soaking period, the core temperatures of the S and C specimens were recorded at 91.5 °C and 111.5 °C, respectively, at 200 °C. At 400 °C, the core temperatures of the S and C specimens were 122.25 °C and 219.34 °C, respectively. At 600 °C, the S specimen exhibited a core temperature of 160.96 °C. The superior thermal insulation properties of alkali-activated slag specimens, as opposed to ordinary Portland cement (OPC) specimens, can be attributed to the formation of microcracks resulting from reactions occurring at elevated temperatures and high alkalinity. Electron microscope images clearly reveal the absence of such fissures in OPC-based specimens. Heat transfer in solid materials is contingent upon phonon movement, specifically the ability of phonons to collide and transfer heat through these collisions. These microcracks serve as scattering sites for impinging phonons, thereby enhancing thermal insulation compared to cement mortar devoid of such obstacles. These obstacles, which may be voids or fillers, act as scatterers for the colliding phonons^90^. Figure 13 also shows that 10 min after reaching a temperature of 400 °C, the core temperature of the S specimens remained relatively stable. A similar trend was observed when the S specimen was subjected to a temperature of 600 °C following a 50-minute heating process. Endothermic reactions, such as capillary evaporation of water and chemically bound water, as well as the decomposition of hydrated compounds, likely account for the stable core temperature.

### Weight loss

**Fig. 14:**
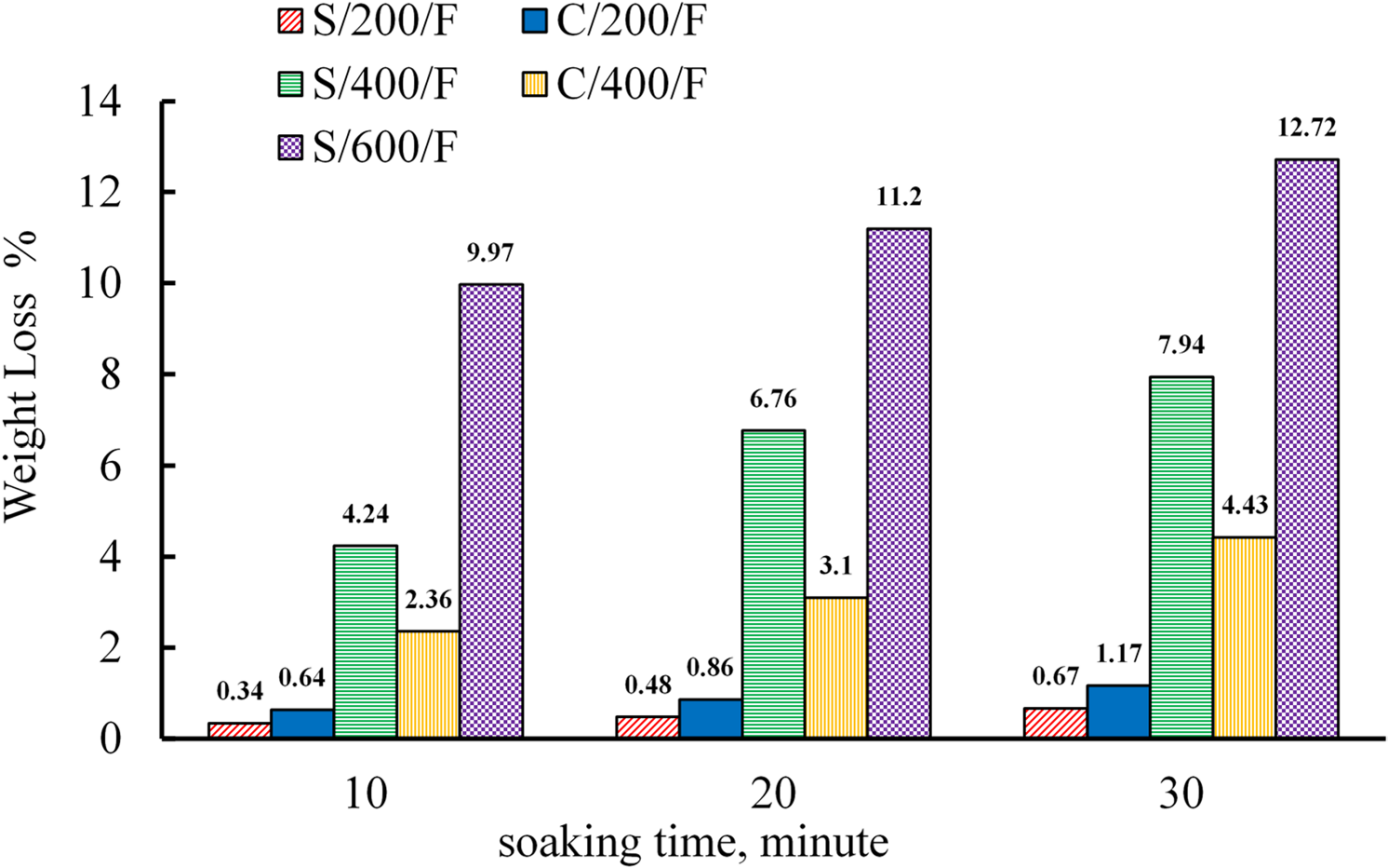
Mass loss of cubic specimens of S and C specimens.

Figure 14 presents the mass loss of S and C specimens due to the evaporation of chemically and physically bonded water as a function of temperature and soaking time for specimens cooled in the furnace. For C specimens, physically bonded water evaporates when heated to 150 °C^91^. The chemical water in the cement matrix evaporates at higher temperatures (above 150–300 °C, depending on the cement composition)^91^. In S specimens, between 105 °C and 300 °C, N-A-S-H and C-A-S-H gels release partly chemically bonded or zeolitic water. As the temperature rises from 300 °C to 500 °C, dehydroxylation of (OH) chains in the geopolymer gel occurs^92,93^. For both S and C mixtures, a slight mass variation was observed at 200 °C, with more pronounced weight loss at 400 °C and 600 °C. These results indicate that the density of S and C specimens decreases with increasing temperature and extended heating periods, as shown in Fig. 14. At room temperature, the average dry unit weight of S and C specimens was 2260 to 2290 kg/m³, respectively. Exposure to 200 °C resulted in a dry unit weight decline of 0.34%, 0.48%, and 0.67% for S specimens and 0.64%, 0.86%, and 1.17% for C specimens at soaking times of 10, 20, and 30 min, respectively. C specimens exhibited higher weight loss than S specimens at 200 °C, likely due to S specimens’ superior thermal insulation, which reduces the evaporation of chemically and physically bonded water during heating. At 400 °C, S specimens experienced weight losses of 4.24%, 6.76%, and 7.94% at soaking times of 10, 20, and 30 min, respectively, while C specimens had weight losses of 2.36%, 3.1%, and 4.43% at the same soaking times. At this temperature, S specimens showed higher mass loss than C specimens, which can be attributed to the disintegration of a larger portion of N-A-S-H and C-A-S-H gels compared to the C-S-H gel. At a temperature of 600 °C, the S specimens exhibited weight losses of 7.97%, 11.2%, and 12.72% at soaking times of 10, 20, and 30 min, respectively.

### SEM analysis

**Fig. 15:**
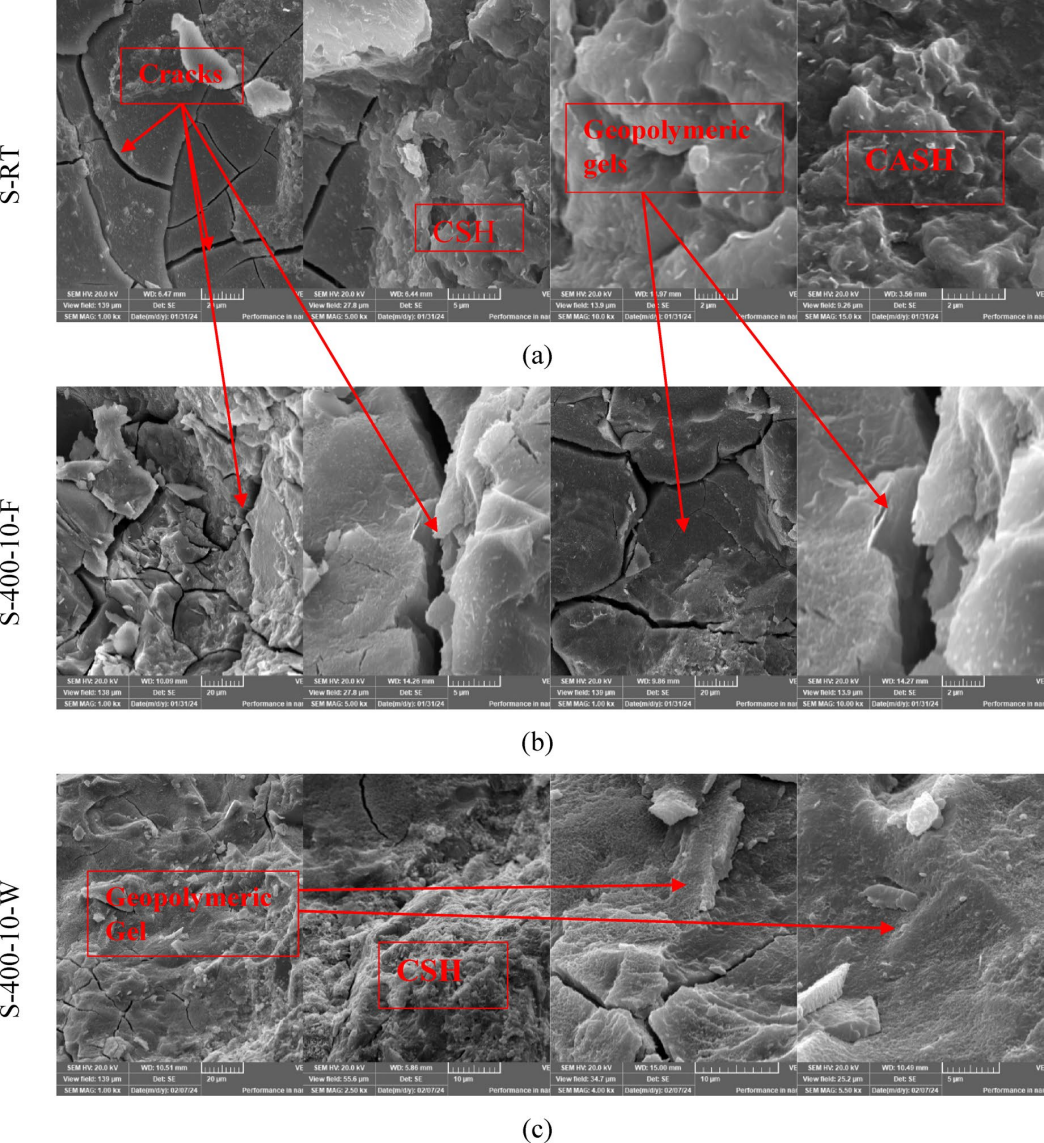

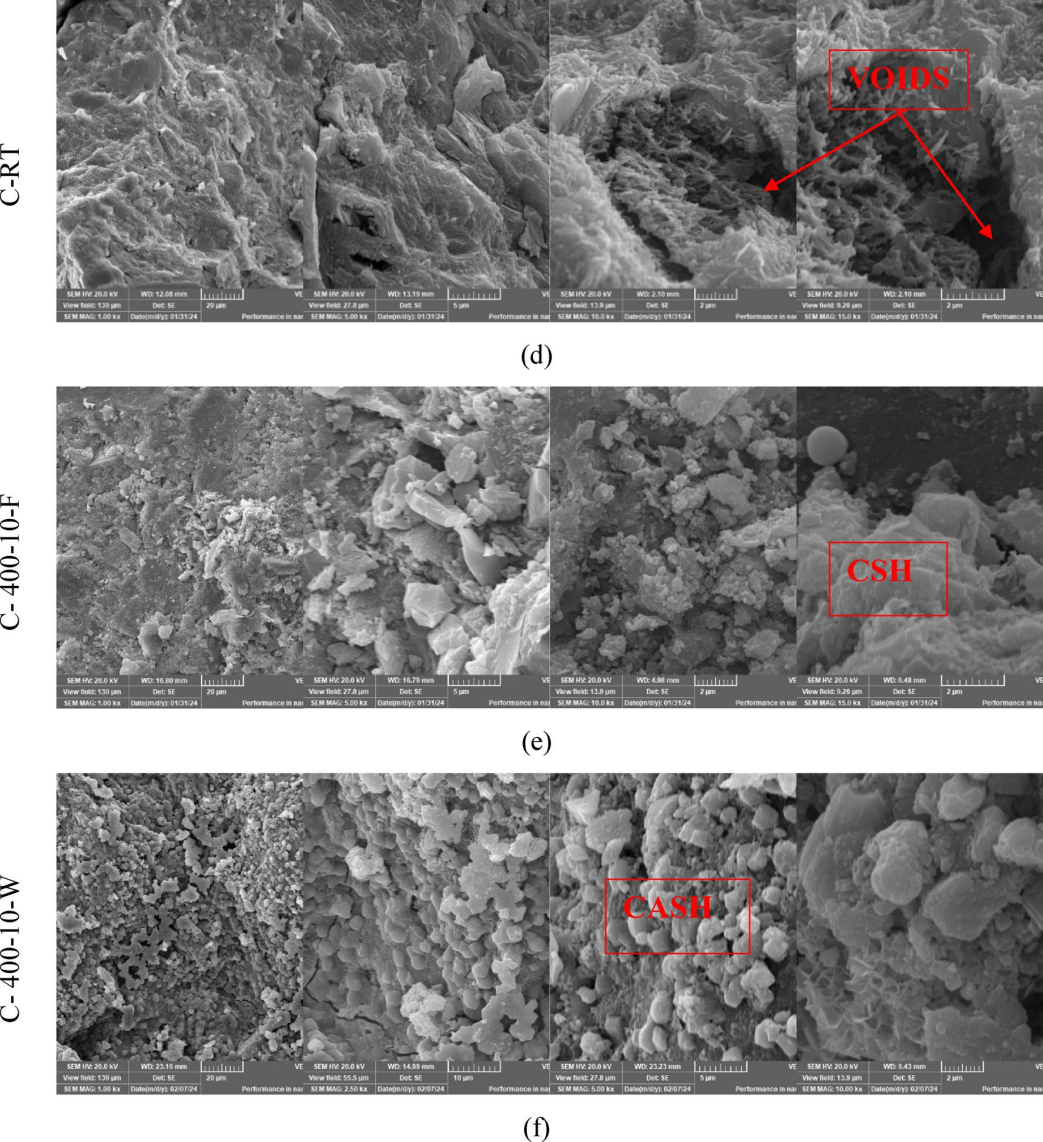
The SEM images of S and C specimens at different magnifications.

Scanning electron microscopy (SEM) images were conducted on selected groups from this study to determine the microstructural alteration of S and C mixtures exposed to varying high-temperature levels for short heating periods and varied cooling regimes. As illustrated in Fig. 15, this mixture is (S, S-400-10-F, S-400-10-W, C, C-400-10-F, C-400-10-W). Figure 15a displays SEM images of S at room temperature, revealing the presence of microcracks. These microcracks result from the rapid reaction that occurs at higher temperatures during the mixing process, as well as the high alkalinity. The presence of microcracks in alkali-activated mixtures improves the connectivity of the void network, allowing water vapors to escape. This helps reduce damage and prevents explosive spalling at high temperatures. In addition, the utilization of a low water-to-binder ratio of 35% has resulted in the detection of unreacted slag particles. As depicted in Figs. 15b and c, exposure to elevated temperatures (400 °C) resulted in noticeable microstructure changes compared to the conditions at room temperature. The changes were manifested through the expansion of the initial microcracks, and the Simultaneous generation of new cracks occurs as a result of both the thermal shrinkage of the matrix and the decomposition of hydrated phases. This leads to the coarsening of the microstructure of the matrix, which in turn reduces the residual compressive strength at temperatures of 400 and 600 °C^45^. Moreover, it has been discovered that the SEM image of S-400-10-W (refer to Fig. 15c) displays a denser and more tightly arranged microstructure compared to S-400-10-F. Consequently, water cooling exhibited a greater remaining compressive strength.

Regarding C specimens, as illustrated in Fig. 15d-f, the SEM images of C specimens at room temperature reveal the presence of microcrystalline and nearly amorphous calcium silicate hydrate (CSH). Additionally, large crystals of calcium hydroxide and black areas indicating the presence of pores were observed. Notably, no microcracks were detected at room temperature for the C mixture, as shown in Fig. 15d.

As seen from Fig. 15e, in comparison to the microstructure at room temperature, the specimens exhibited a denser microstructure after being exposed to high temperatures (T = 400 °C) for 10 min and subsequently cooled in a furnace. As a result, the compressive strength of the specimens that were cooled in the oven was observed to increase by 13.8%. This is due to the fact that the specimens experienced additional hydration as a result of evaporated water during heating. Nevertheless, the specimens exhibited microcracks and voids, which indicate the start of a coarsening of the matrix’s microstructure. This may be due to the presence of pore water pressure and the disintegration of tobermorite and calcium hydroxide^61^. The coarsening of the microstructure and the increase in the number of pores are the reasons why the residual compressive strength showed a decreasing trend with increasing soaking times^67^.

As shown in Fig. 15f, the SEM image C-400-10-W (a sample extracted from an exploded specimen), showed that the formed microcracks were more visible than in the case of the furnace-cooled specimens (see Fig. 15e). These micro-cracks are the result of thermal stresses generated by the sudden cooling, which caused a large difference in temperature between the sample’s surface and its core. A more compact microstructure compared to samples cooled in the furnace was observed due to the rehydration of the dehydrated phases. The formation of more noticeable micro-cracks during cooling in water significantly reduces the tensile strength of the water-cooled samples compared to those cooled in the oven.

**Fig. 16:**
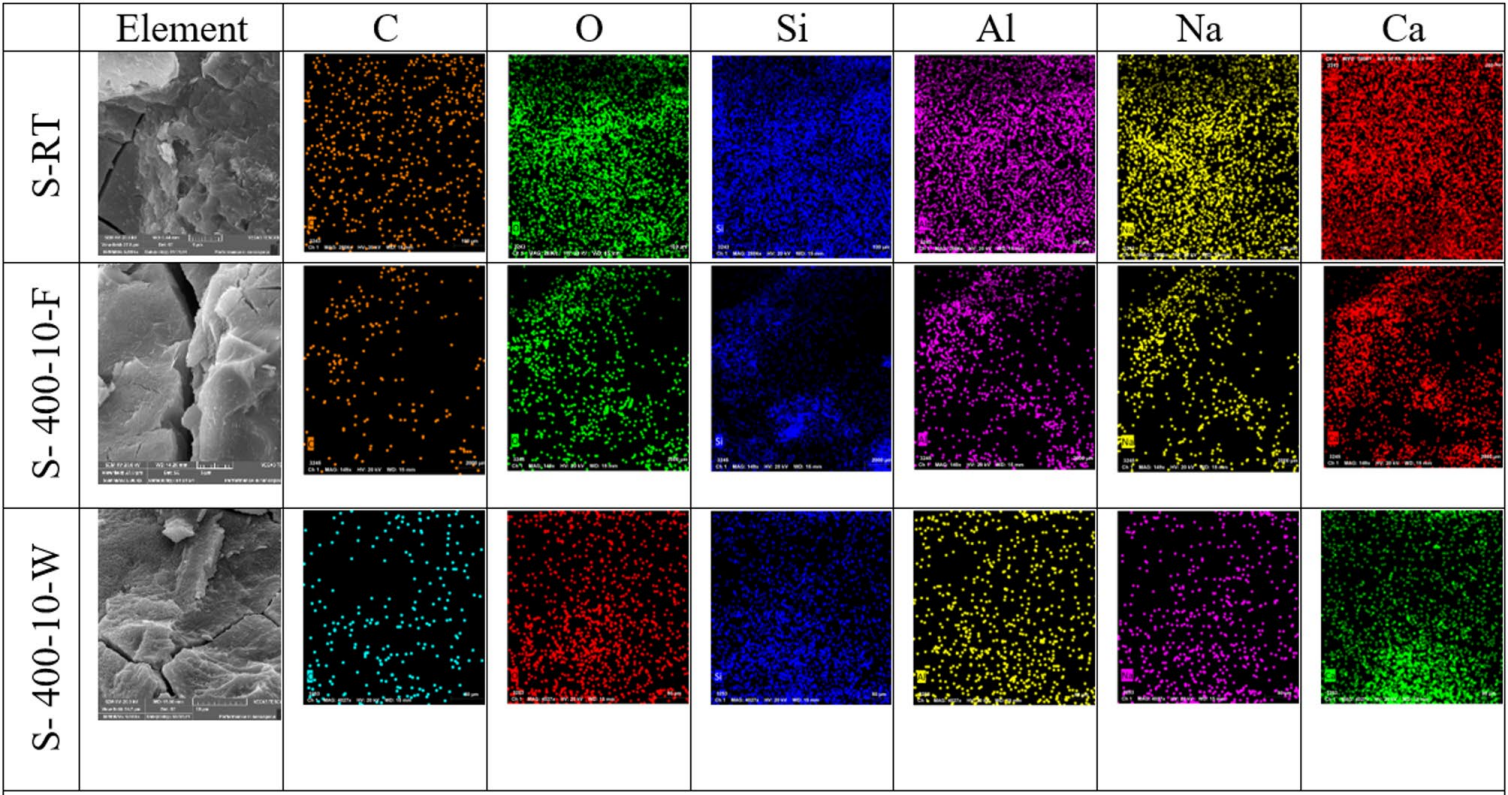
The elemental mapping of S specimens.

**Fig. 17:**
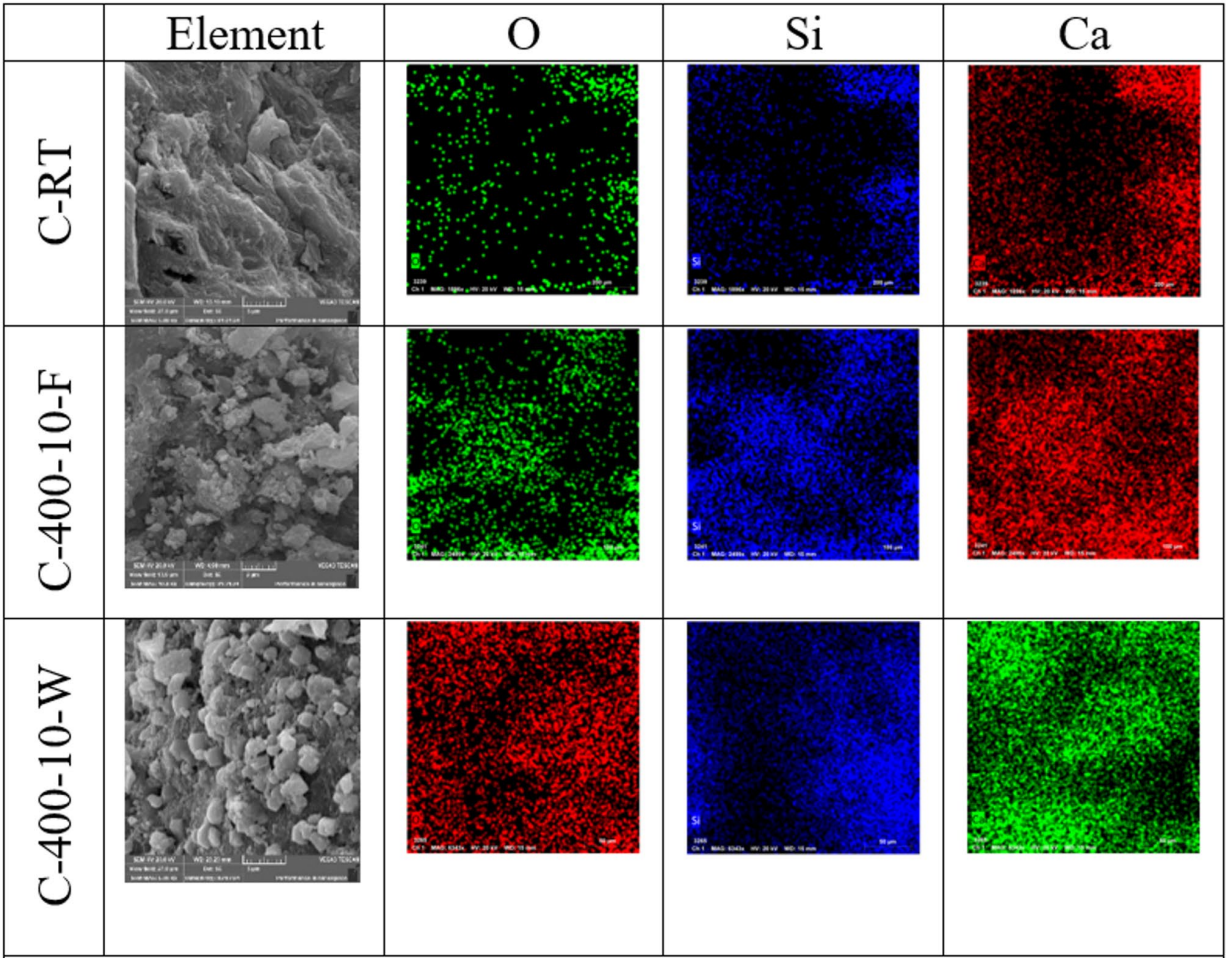
The elemental mapping of C specimens.

Energy Dispersive Spectroscopy (EDS) mapping was employed to conduct a detailed elemental analysis of high-strength AAC (S-mixture) and OHSC (C-mixture), revealing insights into the chemical transformations induced by high-temperature exposure. The EDS mapping results, illustrated in Figs. 16 and 17, indicate distinct elemental distribution patterns between the S-mixture and C-mixture. At room temperature, the S-mixture is characterized by a significant presence of Na, Al, and Si elements derived from the alkaline activators and slag, whereas the C-mixture is dominated by high concentrations of Ca and Si. When exposed to 400 °C for 10 min, the S-mixture exhibited a notable decrease in the intensity of Na and Al, particularly in furnace-cooled specimens, suggesting potential phase reorganization or elemental migration. In contrast, water-cooled specimens (S-400-10-W) maintained a more uniform elemental distribution, indicating the stabilization of amorphous phases. On the other hand, the C-mixture showed consistent Ca distribution, although microstructural cracks became more pronounced, especially in water-cooled samples (C-400-10-W). Microstructural analysis revealed that the S-mixture was more sensitive to thermal treatment, with furnace-cooled samples displaying microcracking and potential strength reduction. Water cooling, however, appeared to enhance phase retention, likely improving compressive strength. In contrast, the C-mixture demonstrated superior thermal stability but experienced issues such as explosion-spalling, coarsening of the microstructure, and increased brittleness under both cooling conditions at 400 °C. These findings highlight the differing thermal responses and structural behaviors of the two materials under high-temperature conditions.

**Fig. 18:**
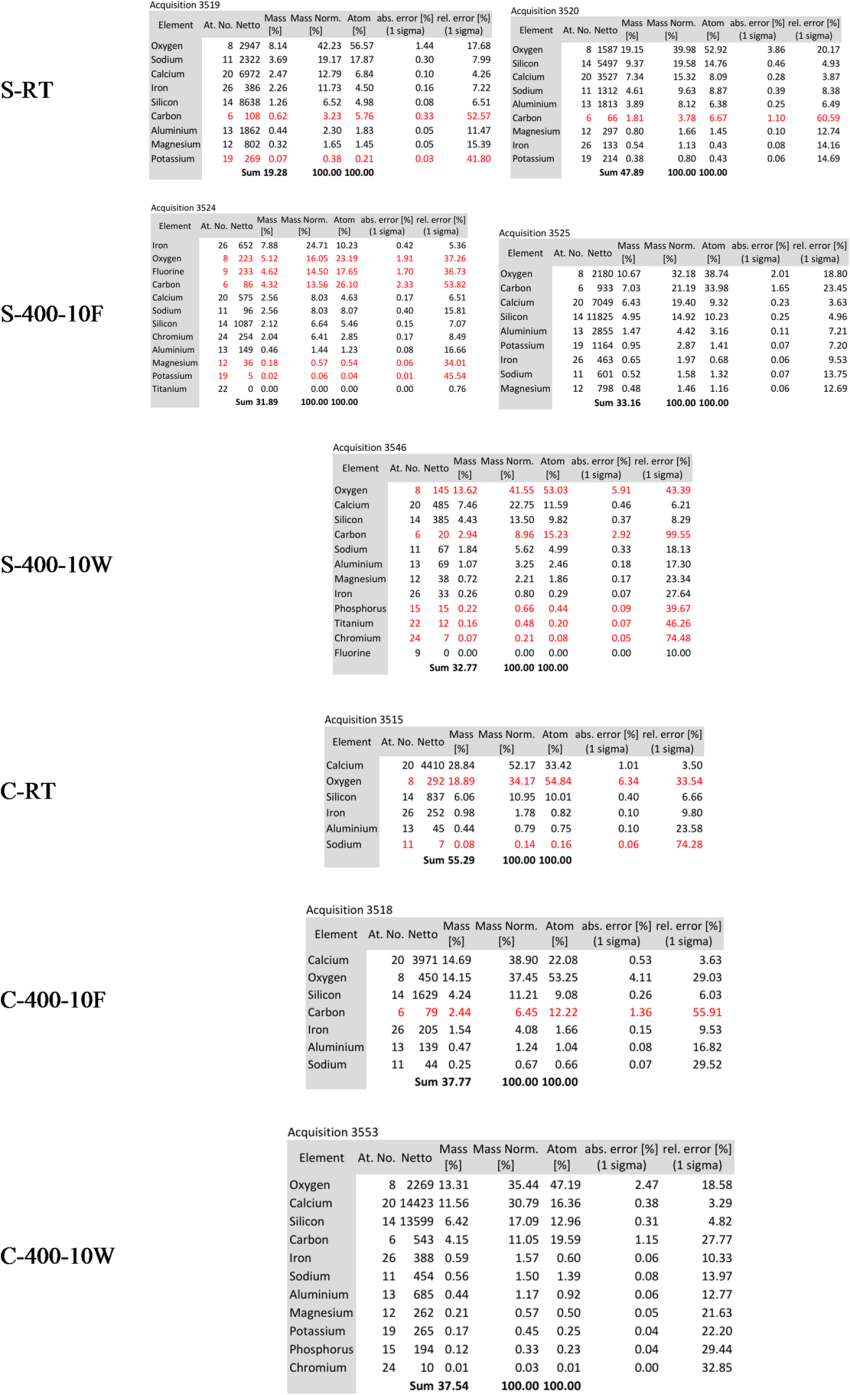
EDS elemental analysis of the S and C specimen.

**Table 12:** The average ratios of ca/si in the matrix of C and S specimens.

ID	Ca/Si
C-RT	3.33
C-400-10-F	2.43
C-400-10-W	1.26
S-RT	1.37 OR 0.55
S-400-10-F	0.85 OR 0.91
S-400-10-W	1.18

From Fig. 18, and Table 12, presented Energy Dispersive X-ray Spectroscopy (EDS) data quantifying the average Ca/Si atomic ratios within the matrix of cement (C) and slag (S) specimens, and provides valuable insights into the compositional changes induced by thermal treatment (400°C) and subsequent curing conditions (Furnace-cooled ‘F’ vs. Water-cured ‘W’). Key observations and interpretations are as follows:

1. **Baseline Composition (RT)**:

- **Cement (C-RT)**: The high Ca/Si ratio (3.33) is characteristic of ordinary Portland cement paste, dominated by calcium-rich phases like portlandite (Ca(OH)_2_) and high-Ca/Si C-S-H phases. This aligns well with established cement chemistry.
- **Slag (S-RT)**: The reported dual values (1.37 OR 0.55) are significant. This pronounced variability strongly suggests inherent heterogeneity within the slag matrix. Slag typically contains a mix of lower Ca/Si C-S-H, hydrotalcite-like phases, and potentially residual glassy phases or crystalline phases like melilite, leading to distinct micro-domains with differing Ca/Si signatures depending on the exact measurement location. The range (0.55–1.37) itself is consistent with the expected lower average Ca/Si of slag systems compared to OPC.

2. **Impact of Thermal Treatment (400 °C) and Cooling**:

- **General Trend (Cement)**: All cement specimens exposed to 400 °C exhibit a substantial decrease in Ca/Si ratio compared to RT (C-RT: 3.33 → C-400-10-F: 2.43 → C-400-10-W: 1.26). This is a critical finding:
  - **Decomposition**: The reduction primarily reflects the thermal decomposition of portlandite (Ca(OH)_2_ → CaO + H_2_O) and potentially the dehydration/dehydroxylation of high-Ca/Si C-S-H phases, leading to a relative enrichment of Si in the remaining matrix.
  - **Cooling Effect**: The significantly lower ratio in the water-cured sample (C-400-10-W: 1.26) compared to the furnace-cooled sample (C-400-10-F: 2.43) indicates that rapid rehydration upon water curing promotes the formation of lower Ca/Si C-S-H phases compared to the slower rehydration/carbonation processes likely occurring during furnace cooling. This suggests water curing post-heat drives a more extensive re-structuring towards denser, silica-richer C-S-H.
  - **General Trend (Slag)**: Slag specimens also show a decrease upon heating (S-RT: 0.55–1.37 → S-400-10-F: 0.85–0.91), though less dramatic than cement and with retained heterogeneity (dual values). This indicates similar dehydration/decomposition processes affecting slag phases (e.g., C-S-H, hydrotalcite, AFm). Notably:
    - **Water Curing Effect (Slag)**: In contrast to cement, water curing the heated slag specimen *increases* the Ca/Si ratio (S-400-10-W: 1.18) compared to its furnace-cooled counterpart (S-400-10-F: 0.85–0.91), and brings it close to the upper end of the RT range. This suggests water curing facilitates significant rehydration, potentially forming new C-S-H with a higher Ca/Si ratio than the dehydrated phases or preferentially reforming certain phases.
  3. **Material-Specific Response**: The data clearly highlights the divergent response of cement and slag matrices to the same thermal and curing regime:
- **Cement**: Shows a monotonic decrease in Ca/Si with thermal treatment, significantly amplified by water curing. Points towards major phase transformations (decay of Ca(OH)_2_, C-S-H modification).
- **Slag**: Exhibits inherent heterogeneity. Thermal treatment reduces Ca/Si on average, but water curing post-heat promotes an *increase* in Ca/Si, suggesting a different rehydration pathway or phase reformation tendency compared to cement. This likely stems from the different initial phase assemblage and chemical potential in slag.

## Conclusion

- **Thermo-Mechanical Degradation**: HSAAM exhibited progressive strength reduction at 400–600 °C, retaining 66.8% compressive strength (52 MPa) after 10 min at 400 °C, while HSCM showed initial strength gains (13.8% at 400 °C) but catastrophic spalling failure beyond 27 min.
- **Cooling Regime Sensitivity**: Water quenching exacerbated strength loss in HSAAM (48.6% at 400 °C) but triggered explosive spalling in HSCM at all exposure durations. Furnace-cooled HSAAM maintained structural integrity, even at 600 °C.
- **Thermal Insulation Superiority**: HSAAM reduced core temperatures by 44% (122.25 °C vs. 219.34 °C for HSCM at 400 °C), attributed to its porous microstructure and microcrack-mediated heat dissipation.
- **Microstructural Stability**: SEM/EDS analysis confirmed HSAAM’s nano-porous matrix facilitated vapor release, preventing pressure buildup. HSCM’s dense microstructure led to internal steam pressure and spalling.
- **Practical Implications**: The absence of spalling in HSAAM up to 600 °C, coupled with retained post-fire mechanical performance, underscores its viability as a fire-resistant material for high-risk structural applications.

## Data Availability

All data generated or analyzed during this study are included in this published article.

## Abbreviations

F_cu_: Residual compressive strength
F_sp_: Residual splitting tensile strength
F_rcu_: Relative residual compressive strength
F_rsp_: Relative residual splitting tensile strength
